# bFGF promotes neurological recovery from neonatal hypoxic–ischemic encephalopathy by IL‐1β signaling pathway‐mediated axon regeneration

**DOI:** 10.1002/brb3.1696

**Published:** 2020-06-11

**Authors:** Zheng Ma, Fang Wang, Lu‐Lu Xue, Ying‐Jie Niu, Yue Hu, Zhang‐Yu Su, Jin Huang, Rui‐Ze Niu, Ting‐Hua Wang, Ying‐Chun Ba, Liu‐Lin Xiong, Xue Bai

**Affiliations:** ^1^ Department of Anatomy Kunming Medical University Kunming China; ^2^ Qingdao Huanghai University Qingdao China; ^3^ Institute of Neuroscience Laboratory Zoology Department Kunming Medical University Kunming China; ^4^ National Traditional Chinese Medicine Clinical Research Base and Western Medicine Translational Medicine Research Center Affiliated Traditional Chinese Medicine Hospital Southwest Medical University Luzhou China

**Keywords:** bFGF‐siRNA, IL‐1β knockout, neonatal hypoxic–ischemic brain damage, neurological recovery, oxygen–glucose deprivation

## Abstract

**Introduction:**

Neonatal hypoxia–ischemic brain damage (HIBD) can lead to serious neuron damage and dysfunction, causing a significant worldwide health problem. bFGF as a protective reagent promotes neuron repair under hypoxia/ischemia (HI). However, how bFGF and downstream molecules were regulated in HI remains elusive.

**Methods:**

We established an in vitro HI model by culturing primary cortical neurons and treated with oxygen–glucose deprivation (OGD). We suppressed the expression of bFGF by using siRNA (small interfering RNA) interference to detect the neuronal morphological changes by immunofluorescence staining. To determine the potential mechanisms regulated by bFGF, the change of downstream molecular including IL‐1β was examined in bFGF knockdown condition. IL‐1β knockout (KO) rats were generated using CRISPR/Cas9‐mediated technologies. We used an accepted rat model of HI, to assess the effect of IL‐1β deletion on disease outcomes and carried out analysis on the behavior, histological, cellular, and molecular level.

**Results:**

We identified that OGD can induce endogenous expression of bFGF. Both OGD and knockdown of bFGF resulted in reduction of neuron numbers, enlarged cell body and shortened axon length. We found molecules closely related to bFGF, such as interleukin‐1β (IL‐1β). IL‐1β was up‐regulated after bFGF interference under OGD conditions, suggesting complex signaling between bFGF and OGD‐mediated pathways. We found HI resulted in up‐regulation of IL‐1β mRNA in cortex and hippocampus. IL‐1β KO rats markedly attenuated the impairment of long‐term learning and memory induced by HI. Meanwhile, IL‐1β^−/−^ (KO, homozygous) group showed better neurite growth and less apoptosis in OGD model. Furthermore, serine/threonine protein kinase (AKT1) mRNA and protein expression was significantly up‐regulated in IL‐1β KO rats.

**Conclusions:**

We showed that IL‐1β‐mediated axon regeneration underlie the mechanism of bFGF for the treatment of HIBD in neonatal rats. Results from this study would provide insights and molecular basis for future therapeutics in treating HIBD.

## Graphical abstract

Hypoxic‐ischemic brain damage (HIBD) can lead to serious neuron damage and dysfunction, and cause serious world health problems. BFGF has been reported as a protective agent to promote neuronal repair under hypoxia/ischemia (HI). We found that the axon regeneration mediated by IL‐1 β is one of the mechanisms of bFGF in the treatment of hypoxic‐ischemic encephalopathy. The results of this study will provide theoretical and molecular basis for the future treatment of HIBD.

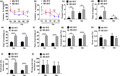

## 1 INTRODUCTION

Neonatal hypoxic–ischemic brain damage (HIBD) is a relatively common malignant complication caused by clinical perinatal asphyxia in infants and young children (Ornitz & Itoh, 2001; Wang et al., 2015), which occurs in 1–6 of every 1,000 live term births (Gu et al., 2016). Statistics suggests that approximately 40% of the affected infants die in the neonatal period and an additional 30% have lifelong neurological deficits including cerebral palsy, epilepsy, and cognitive disabilities (Rocha‐Ferreira & Hristova, 2016), although the best care is given. Therefore, HIBD results in substantial socio‐economic burden of the individual, family, and healthcare system (Han, Ding, Xu, Liu, & Feng, 2016), presenting as a major public health issue in the word. Many neuroprotective therapies appear promising in animal experiments, most of them are unreliable or ineffective in human patients with hypoxic–ischemic encephalopathy (HIE) (Sekiguchi et al., 2018). Currently, there is no effective treatment to minimize brain disease induced by hypoxia–ischemia (HI) in clinical (Liu, Zhu, Zou, Wang, & Fu, 2015). Therefore, it is urgent to explore new mechanism so as to further optimize HIE treatment.

It is well known that HIBD is a progressive and evolving process, involving a variety of biochemical mechanisms and pathways underlying both early and delayed injury (Grow & Barks, 2002). However, available molecule for targeting therapy is still missing. Basic fibroblast growth factor (bFGF) is one of neurotrophic factor members consisting a family of growth‐related factors, essential for angiogenesis, wound healing, embryonic development, and various endocrine signaling pathways, expressing in different development stage ranging from nematodes to humans (Ornitz & Itoh, 2001). As one of the most important FGFs, bFGF shares vital biological functions, including proliferation, differentiation, migration, and apoptosis, and also has been reported in promoting nerve regeneration after HI insult. The multifunction of bFGF was originated from the complication of the signal transduction of bFGF (Kuhn et al., 2012). Yin XJ found that bFGF is effective for nerve recovery and inhibition of neuronal apoptosis after HIBD (Yin, Liu, Luo, Long, & Feng, 2009), indicating bFGF could serve as a useful treatment for HIBD under clinical settings. However, the detailed molecular mechanism underlying bFGF in regulating neural regeneration and related cytokine secretion remains elusive.

As far as we know, there has been no literature on the evidence for the protective effect of bFGF on neurons after HIBD and its molecular mechanisms. As a well‐known model of cerebral HI in vitro, OGD provided a useful method for us to determine the role of bFGF and related molecular network (Chen et al., 2019). In this study, we aimed to study the role of bFGF in maintaining normal number and morphology of cortical neuron cells by establishing an in vitro model to mimic HI process in vivo and to determine relevant molecular targets. Then, we use transgenic rats to further explore the molecular mechanism. Results from this study would provide insights and molecular basis for future therapeutics in treating HIBD.

## 2 MATERIALS AND METHODS

### 2.1 Animals

One‐day‐old Sprague Dawley (SD) rats (both male and female were acceptable) weighing 5.5 g were provided by Experimental Animal Center of Kunming Medical University, Kunming, China. Rat pups were randomly divided into eight groups: 1. normal group; 2. reagent group; 3. negative control (NC) group; 4. bFGF‐si group; 5. OGD group; 6. OGD + reagent group; 7. OGD + NC group; 8. OGD + bFGF‐si group. In addition, IL‐1β knockout rats were constructed by Cyagen Biosciences Inc. KO rats were bred and genotyped. Aseptic environment was maintained in all rats during surgical procedures. All of the experiments conformed to the Guide for the Care and Use of Laboratory Animals published by the US National Institutes of Health. This study was conducted in accordance with the principles of the Basel Declaration and recommendations of the Ethical Committee of Kunming Medical University (reference number: kmmu 2018024). All animals were housed in cages with a 12‐hr light/dark cycle and had free access to food and water.

### 2.2 Primary cortical neuron culture

Primary cortical neuron cultures were extracted from the cerebral cortex of 10 one‐day old Sprague Dawley rats. They were deeply anesthetized by isoflurane and euthanized by decapitation. The cortex tissue was gently separated with the white matter removed, and the meninges were gently peeled. Cortices were cut into small pieces, dissociated into a cell suspension, and plated in coverslips (24 mm × 24 mm) with poly‐L‐lysine. The isolated cells were then cultured in a neurobasal medium (Gibco, USA) containing 2% B27 (Gibco, USA), 0.5 mM glutamine, 50 U/ml penicillin and 50 μg/ml streptomycin. Afterward, the harvested neurons were incubated in 37°C chamber with 5% CO_2_. In addition, the neurobasal medium was firstly replaced after 24 hr of incubation and then half of the medium was replaced every three days. After the primary cerebral cortical neurons grew in well for 5 days (d), cells were divided into eight groups as above. The NC group was transfected with a negative control siRNA to ensure specificity. The reagent group was transfected with a transfection reagent to rule out the possible effects of the reagent on the cells. Cells in the OGD + Negative Control and OGD + bFGF‐si groups were given the neurobasal medium with siRNA, without penicillin/streptomycin for 12 hr and then cultured in normal neurobasal medium. 3 days after transfection, supernatants were removed and cells were collected with TRIzol for total RNA extraction and the expression levels of bFGF and IL‐1β mRNA were analyzed by Q‐PCR.

### 2.3 PC12 cell culture

PC12 cells were purchased from American Type Culture Collection (ATCC, United States) and cultured according to the guidelines recommended by ATCC. We quickly thawed PC12 cells in 37°C water. The cells were seeded in 25 cm^2^ polystyrene flasks with 4.5 g/L glucose in Dulbecco's modified Eagle Medium (DMEM) (×1) (Hyclone) supplemented with 10% heat inactivated fetal bovine serum (Gibco), 50 U/ml penicillin, and 50 μg/ml streptomycin. Cells were incubated at 37°C under a humidified atmosphere of 95% O_2_ and 5% CO_2_. Culture medium was replaced every 48 hr, and cultures were split at a ratio of 1:6 once a week. We used PC12 cells for validation of bFGF siRNA fragments.

### 2.4 Cell counts

We determined the density of the inoculated cells by cell counting. First, 90 μl of DMEM and 10 μl of cell suspension were added to a 1.5 ml EP tube. Then, 8 μl of the cell suspension was added to the blood cell counting plate, and the number of cells in the four quadrants was counted by a microscope. The final cell count was calculated as follows: total number of four quadrants/4 × dilution factor × 10^4^ cells/ml. We inoculated a cell density of 5 × 10^5^/ml in each well of a 6‐well plate.

### 2.5 Screening for effective fragment of siRNA

bFGF gene sequence was blasted from NCBI database, then three 19‐nucleotide sequences were designed corresponding to the bFGF reference sequence (NCBI, NM_001361665.1/NM_002006.5). One scrambled siRNA was designed and purchased from RuiBo Company Guangzhou, China, as a negative control. To filter the most effective fragment, cultured PC12 cells were randomly divided into normal group, NC group, reagent group, bFGF‐si‐F1 group, bFGF‐si‐F2 group, bFGF‐si‐F3 group and CY3 group. The procession was performed according to the manufacturer's standard protocol. The specific operation steps as described “siRNA Transfection.” The siRNA against bFGF with the best knock down effect was selected for following experiments.

### 2.6 siRNA transfection

Primary neurons were split into 6‐well plates at day 5 after culture, divided into eight experimental groups (*n* = 10 wells/each), and extra wells were prepared for CY3 transfection to determine transfection efficiency. When cell density reached approximately 80% confluency, cells were subject to siRNA transfection using the riboFECTTM CP transfection reagents according to manufacturer's instruction without penicillin/streptomycin. In brief, each well of 6‐well plates was added with a mixture of 60 μl 1 × buffer and 5 μl siRNA/CY3 or 6 μl siRNA reagent. The plates were placed in 5% CO_2_ incubator at 37°C for 24 hr to 96 hr, with media replaced at 12 hr post‐transfection. Fluorescent images were obtained from Leica AF6000 cell station after 48 hr, and cell numbers were calculated based on images at high power field (200 ×), while transfection efficiency was determined by above 80% of red channel positive cells/total cells.

### 2.7 Oxygen–glucose deprivation

Seven days after plating, the primary cortical neurons were washed twice with glucose‐free DMEM (Gibco) and the medium was replaced with glucose‐free DMEM containing L‐glutamine (Gibco) that had been deoxygenated with an anaerobic gas mixture (95% N_2_ – 5% CO_2_) for 30 min before use. The cells were then placed in a hypoxic chamber, flushed with the anaerobic gas mixture (95% N_2_ – 5% CO_2_) and incubated at 37°C for 3 hr to establish cell model of oxygen–glucose deprivation (OGD). After OGD, the cells were incubated under normal culture conditions for 0 hr or 24 hr. Neurons in the normal group were treated without OGD exposure.

### 2.8 Methyl thiazolyl tetrazolium analysis

The cell viability of neurons was detected using methyl thiazolyl tetrazolium (MTT) assay was performed at 24 hr after OGD induction (Zhang et al., 2019). Briefly, neurons were seeded in 96‐well plates precoated with poly‐L‐lysine. At 24 hr after OGD, cells were washed with PBS after the medium was removed. Neurons were incubated in serum‐free medium containing MTT (20 μl, 5 g/L, Beyotime, Shanghai) solution for 4 hr at 37°C in the dark. Then, the MTT solution was removed and 100 μl of DMSO was added to each well to dissolve the formazan crystals. The optical density values were determined at a wavelength of 562 nm using a microplate plate reader (Bio‐Rad, Hercules). Cell viability was calculated as the ratio relative to the control.

### 2.9 Immunofluorescence (IF) staining

To examine the changes of cell number and morphology following transfection and knockdown efficiency of siRNA, comparative analysis of immunofluorescence staining was performed in the primary cortical neurons. Briefly, neurons were washed with PBS for three times (5 min for each time) and then fixed in 4% paraformaldehyde (pH 7.4) for 10 min. The neurons were then blocked with 5% normal goat serum for 30 min and incubated overnight at 4°C in a humidified chamber with the following primary antibody: TUJ1 (Rabbit, Abbkine, 1:100). After being washed for three times, the neurons were incubated with fluorescence‐labeled goat secondary antibody: Alexa Fluor 488 (anti‐rabbit, Abbkine, 1:200) for 1 hr at 37°C in dark. The neurons were next washed with PBS for 3 times for five minutes each time before the nuclei were stained by DAPI (Beyotime). Cell image was collected using a confocal microscope (Zeiss LSM). We have measured five images of each sample (*n* = 10) in each group and used the average of the number of cells in five images. The neurite lengths and neuron surface (cell body) of TUJ1^+^ cells were quantified by Image Pro Plus 6.0 software (Media Cybernetics) at 24 hr after OGD induction.

### 2.10 Terminal‐deoxynucleoitidyl transferase mediated nick end labeling (TUNEL) staining

The apoptosis rate of primary cortical neurons was measured using the TUNEL assay. The procedure before incubating the antibody was the same as the IF method. Then, the neurons were incubated with TUNEL reaction mixture (In situ Cell Death Detection Kit; Roche) at 37°C for 1 hr under humidified conditions and further washed with PBS. Finally, the neurons were stained with DAPI and the images were acquired using Leica AF6000 cell station.

### 2.11 Determination of nitrogen oxide (NO) generation in primary cortical neurons

Nitrogen oxide were used as markers of oxidative stress and to detect the extent of brain damage. The NO generation was analyzed as previously described with minor modification (Yu, Lin, Zhang, & Guo, 2004). The levels of NO in primary cortical neurons were measured following the relevant quantitative kit instructions (Jiancheng, Nanjing, China). The neurons were pulverized in liquid nitrogen and suspended in homogenization buffer (NaCl 0.1 M, Tris‐HCl 0.01 M, pH 7.6, egtazic acid 1 mM, aprotinin 1 mg/L, and PMSF 100 mg/L). After centrifugation, 50 µl Griess reagent (equal volume of 1% sulfanilamide in HCl 0.1 M and 0.1% N‐ [−1‐naphthyl‐ethylenediamine dihydrochloride]) was added to 50 µl of suspending media. Nitrite concentration was determined by spectrophotometry (560 nm) from a standard curve (0–100 mM) derived from NaNO_2_ (Beyotime Biotechnology). The data were expressed as mean ± *SD* (nitrite) in µM.

### 2.12 Neonatal hypoxia–ischemia rat model establishment

Seven‐day‐old rats (12–15 g) were anesthetized in a glass vial filled with 0.3% isoflurane, and removed and placed in a supine position. The right common carotid artery was exposed through an incision in the middle of neck which was then ligated with electro coagulation (Spring Medical Beauty Equipment co., LTD, Wuhan, China). After the wound was sutured, the rats were transferred to 37°C incubator (8% O_2_, 92% N_2_) to induce hypoxia for 2 hr. The rats in the Sham‐operated group only exposed the right common carotid artery and did not undergo ischemia and hypoxia. After hypoxic exposure, the animals were sent to their cages to recover from surgery.

### 2.13 Tissue harvest

The rats at 24 hr after HI were anesthetized with isoflurane and then fixed in a supine position on the dissection table. The needle was inserted into the aorta from the left ventricle of the rat and perfused with 0.9% saline. After the whole body blood of the rat was replaced, 4% paraformaldehyde was perfused until the body became hard. Subsequently, the entire brain was quickly moved into a 4% paraformaldehyde solution for more than 24 hr to prepare for paraffin embedding. In addition, the tissues required for qRT‐PCR and WB experiments did not need to be perfused with 4% paraformaldehyde, and the right cortex, left cortex, right hippocampus, left hippocampus, lung, and heart were taken separately. The tissue was harvested and stored in liquid nitrogen.

### 2.14 Hematoxylin and eosin (HE) staining

The brain tissues fixed with 4% paraformaldehyde were chopped into 0.5 cm × 0.5 cm × 1 cm pieces. Then, brain tissues were dehydrated until transparent, immersed in wax, and embedded in paraffin. The paraffin‐embedded tissues were cut into 5 μm‐thick coronal sections using a Rotary Microtome YD‐1508R (Jinhua YIDI Medical Appliance Co., Ltd). Then, the paraffin sections were routinely stained with hematoxylin and eosin (HE) (BL702A, Biosharp; C0105‐2, Beyotime). Next, the stained sections were using 50× and 200× OLYMPUS BX43 microscope (Olympus Co.). After diameter was measured, the thickness and average cell nucleus size of prefrontal cortex area and CA1, CA2, CA3, and DG area in the hippocampus was measured using image J (NIH).

### 2.15 Nissl staining

Rats were transcardially perfused with saline followed by 4% paraformaldehyde. Then, the entire brain was removed and placed in 4% paraformaldehyde fixative. After fixation for more than 24 hr, brain was embedded in wax and cut into 5 μm‐thick coronal sections. Subsequently, paraffin sections were dewaxed and stained with toluidine blue (Beyotime Biotechnology). Three sections were randomly selected from each rat, and the total number of cells in the prefrontal cortex area and hippocampus was observed and counted using a 200× field OLYMPUS BX43 microscope. ImageJ was used to count the number of nerve cells. Eventually, the number of neuron cells was calculated [(average neuron number in sections of HI − number of neurons in sections of treated Sham group)/average neuron number in Sham sections × 100].

### 2.16 Construction of IL‐1β knockout rats

To investigate the function of IL‐1β in rats with HIE, we constructed IL‐1β KO rats by using CRISPR/CAS9 technology. The rats’ IL‐1β gene (GenBank accession number: NM_031512; Ensemble: ENSRNOG00000004649) is located on rat chromosome 3. Seven exons have been identified with the ATG start codon in exon2 and TAA stop codon in exon7. Exon4 was selected as the target site. Cas9 plasmid and sgRNA vector (Figure S1a) were transcribed in vitro synthesized Cas9 mRNA and small guide RNA (sgRNA). Subsequently, a mixture of Cas9 mRNA (20 ng/μL) and sgRNA (10 ng/μL) was microinjected into the fertilized eggs of *SD* rats using prokaryotic injection to obtain IL‐1β KO rats. The genetically modified rat target amplification products are present in the F1 generation. The results of PCR screening (TaKaRa marker DL2000) showed that the band from knockout rats (homozygous) was at around 630 bp (−/−, blue arrow), the band from wild‐type rats was at around 1195bp (+/+, yellow arrow), and two bands (±, red arrow) appear simultaneously in heterozygous rats (Figure S1b). Finally, IL‐1β KO rats were used to study the function of IL‐1β by mass breeding.

### 2.17 Primary cultures of cortical neurons from IL‐1β KO rats

Briefly, tissues of cerebral cortex were harvested from brains of Sprague Dawley IL‐1β KO and wild‐type (WT) rat embryos (aged 1 day). The specific procedure was the same as the section of “Primary Cortical Neuron Culture.”

### 2.18 Morris water maze (MWM)

Morris water maze (MWM) was first established by neuroscientist Richard G. Morris in 1981 in order to test ability of spatial memory and long‐term spatial memory (Morris, 1981). To demonstrate whether the HI model was successful and affected the long‐term spatial memory of rats, the MWM was performed test at one month (1 m) and two months (2 m) after HI surgery in rats. The MWM consisted of a circular pool with a diameter of 900 mm and 50 cm height filled water (22 ± 1.5°C) to 40 cm deep. The platform was placed below the surface of water at 1 cm and add ink to prevent rats from seeing the platform. The pool was divided into four quadrants, and the platform was placed in the center of one quadrant. As previously described (Vorhees & Williams, 2016), the rat was asked to find the location of a hidden platform below the surface of the water and experienced four trials per day from different release positions for six consecutive days. The experiments were divided into two stages: directional navigation and space exploration. (a) Directional navigation: During the training phase of the first 5 days, if the rat failed to escape on the platform within 90 s, it was guided to climb on platform and stay on the platform for 10 s. Then, the video‐tracking system was used to record the latency of finding the platform. (b) Space exploration: During the acquisition phase, the swimming time in target platform was recorded. On the sixth day of test, the platform was removed and the rat was allowed to search the maze for 90 s, whereas the number of platform area crossings was measured. In addition, the MWM testing environment decreases odor trail interference. This test was widely used in neurobiology and neuropharmacology research of spatial learning and memory.

### 2.19 Open field test

Open field test was used to assess the exploratory activity and emotional changes caused by exposure of rats to new environments at 1 month (1 m) and 2 months (2 m) after HI (Molina, Capani, & Guelman, 2016). Rats were placed in a closed square area (length: 100 cm; width: 100 cm; depth: 40 cm) for 10 min and rat activity was recorded using Smart 3.0 video tracking software (Switzerland). A 100 cm (length) × 100 cm (width) square area (Xinruan) was digitally divided into 25 quadrants of the same size. The system automatically recorded the time (s) that rat spent standing and grooming and analyzed the data.

### 2.20 Y maze test

Short‐term spatial working memory was evaluated by recording spontaneous alternation behavior during a single session in the Y maze (made with three arms, 50 cm long, 10 cm width, 25 cm arm height, 120° separate) positioned at exactly the same location for all procedures (Sarnyai et al., 2000). Rats were tested for Y maze at one and two months after suffering HI. Three arms were named as the starting arm, the food arm, and the wrong arm. In short, the rats were fasted for 1–2 days to achieve a 20% weight loss before the test. Then, each rat was placed at the end of the starting arm and moved freely through the maze within 10 min looking for food placed on the food arm. Each rat was trained three times. Finally, the food from the food arm was removed and the rats were formally tested. An arm choice was considered only when both fore paws and hind paws fully entered the arm. The system automatically (Xinruan) records the number and time of each fasting rat stays in each arm within 5 min. The Y maze was cleaned after each test with 80% ethanol to minimize odor cues. The correct rate was calculated as number of food arms/total number of three arms × 100. The error rate was calculated as number of wrong arms/total number of three arms × 100.

### 2.21 RNA extraction and quantitative real‐time PCR (Q‐PCR)

Each 1 × 10^6^ cells or 50 mg tissues were mixed with 1ml TRIzol (Thermo Fisher Scientific) reagent followed by RNA extraction process according to manufacturer's direction. The extracted RNA was reverse transcribed into cDNA using Revert Aid TM First Strand cDNA Synthesis Kit (Fermentas). We performed Q‐PCR of bFGF by using All‐in‐One TM q‐PCR Mix (GeneCopoeia) on an ABI 7500HT System (Applied Biosystems) to determine the expression levels of bFGF, IL‐1β and AKT1. β‐actin was used as endogenous controls. The sequences of the primers are listed in Table 1. The Q‐PCR program was 95°C 5 min, 45 cycles of 95°C 15 s, 75°C 30 s, 60°C 30 s, and 60°C 30 s. The final PCR products were analyzed by electrophoresis using 1% agarose gel and visualized by GoldView with Alpha Innotech (Bio‐Rad) to determine if the positive bands were obtained. Lastly, the total amount of the template was determined according to the standard curve with a 2^−△△^ *^C^* ^t^ method.

**Table 1:** Primer sequences

Genes	Forward (5′−3′)	Reverse (5′−3′)
bFGF	TCCCAAGCGGCTCTACT	ACTCCAGGCGTTCAAAGA
IL−1β	GAGCTGAAAGCTCTCCACCT	TTCCATCTTCTTCTTTGGGT
AKT1	AGTCCCCACTCAACAACTTCT	GAAGGTGCGCTCAATGACTG
β‐actin	GAAGATCAAGATCATTGCTCCT	TACTCCTGCTTGCTGATCCA

### 2.22 Western blotting

Cells were homogenized with RIPA buffer (50 mM Tris‐HCl (pH 7.4), 150 mM NaCl, 0.1% SDS, 2 mM EDTA, 1% Na‐deoxycholate and 1% NP‐40) supplemented with cocktail protease inhibitor (1:500; Roche, Castle Hill, NSW, Australia). All homogenates were centrifuged at 15,000 RCF for 30 min at 4°C to collect the supernatants and total protein concentration was assessed by BCA assay (Beyotime Biotechnology). After boiling at 100°C for 5 min, 12% SDS‐polyacrylamide gels were used to separate 30 µg protein per well followed by a semi‐dry transfer for 20 min. After blocking with 5% nonfat milk, membranes were probed with primary antibodies (IL‐1β, USCN, cat# PAA563Rb51, rabbit, 1:2,000; AKT, Bioss, cat#bs‐0115R, rabbit, 1:500; phospho‐AKT, Bioss, cat#bs‐5194R, rabbit, 1:500) overnight at 4°C. β‐actin (Abbkine, cat# A01010, mouse, 1:2,000) was used as a loading control. After incubation with appropriate secondary antibodies (HRP, Goat Anti‐Mouse IgG, cat# A21020, 1:5,000; HRP, Goat Anti‐Mouse IgG, cat# A21010, 1:5,000) for 1 hr at room temperature, protein bands were visualized and analyzed using a chemiluminescent imaging system (Bio‐Rad).

### 2.23 Statistical analysis

Sigma plot software SPSS version 20.0 (SPSS Inc.) was used to perform data analysis, and all data were expressed as mean ± standard deviation (*SD*). For multiple group comparison, ANOVA with Tukey's post hoc multiple comparisons was applied. Standard *t* test was used to analyze the data between two groups. Repeated measures was used to repeated measure. *p* < .05 was considered to be statistically significant and the significance level is shown by *p < .05; **p < .01; ***p < .001.

## 3 RESULTS

### 3.1 OGD induced bFGF expression in primary cortex neurons in vitro

**Figure 1:**
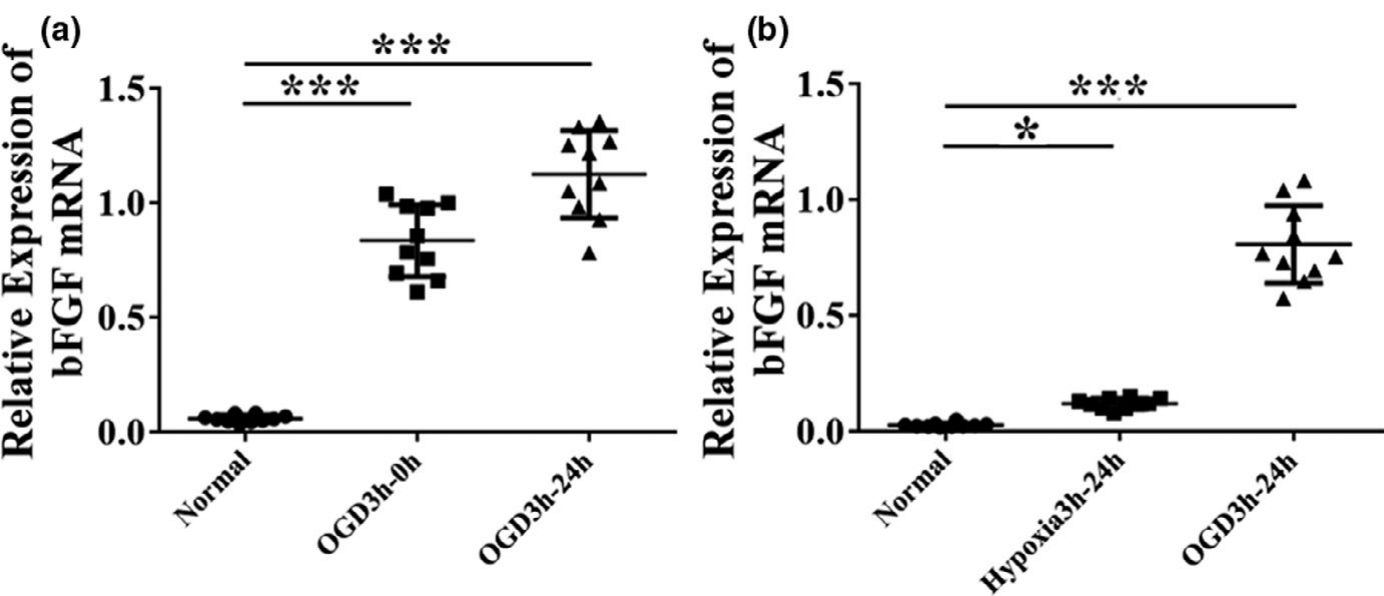
OGD induces bFGF mRNA expression in vitro. (a) Relative expression of bFGF mRNA among normal, OGD3h‐0h, and OGD3h‐24h groups. (b) Relative expression of bFGF mRNA among normal, hypoxia 3h‐24h, and OGD3h‐24h groups. mRNA expression was plotted as 2^−ΔΔ^ *^C^* ^t^. *n* = 10/group. Ordinary one‐way ANOVA, with LSD test was performed. *p < .05; ***p < .001. Abbreviation: OGD3h‐0h, primary cortical neurons were reoxygenated immediately after 3 hr of OGD treatment; OGD3h‐24h, primary cortical neurons were reoxygenated at 24 hr after 3 hr of OGD treatment; Hypoxia 3h‐24h, primary cortical neurons were reoxygenated at 24 hr after 3 hr of hypoxic treatment. OGD, oxygen–glucose deprivation

Given that the HIE pathogenesis is difficult to study in vivo, we utilized the cultured primary neurons subject to OGD to mimic the HI process and examined the expression levels of bFGF. We identified that the mRNA levels of bFGF were significantly elevated immediately after OGD treatment for 3 hr, as well as 24 hr post OGD back in reoxygenation in comparison to the non‐OGD treatment normal (*p* < .001, Figure 1a). Due to the fact that OGD treatment included both deprivation of oxygen and glucose, next we evaluated whether hypoxia was able to induce bFGF expression. We established a hypoxic model where cells underwent hypoxia for 3 hr without depriving glucose and then restored oxygen for 24 hr. We identified that hypoxia only could slightly induce transcriptional bFGF expression (*p* < .05, Figure 1b), which could be magnified by depriving glucose at the same time (*p* < .001, Figure 1b). These results suggested that bFGF expression levels were stimulated in primary cortical neurons after OGD exposure.

### 3.2 Successful knockdown of bFGF in primary cortical neurons

**Figure 2:**
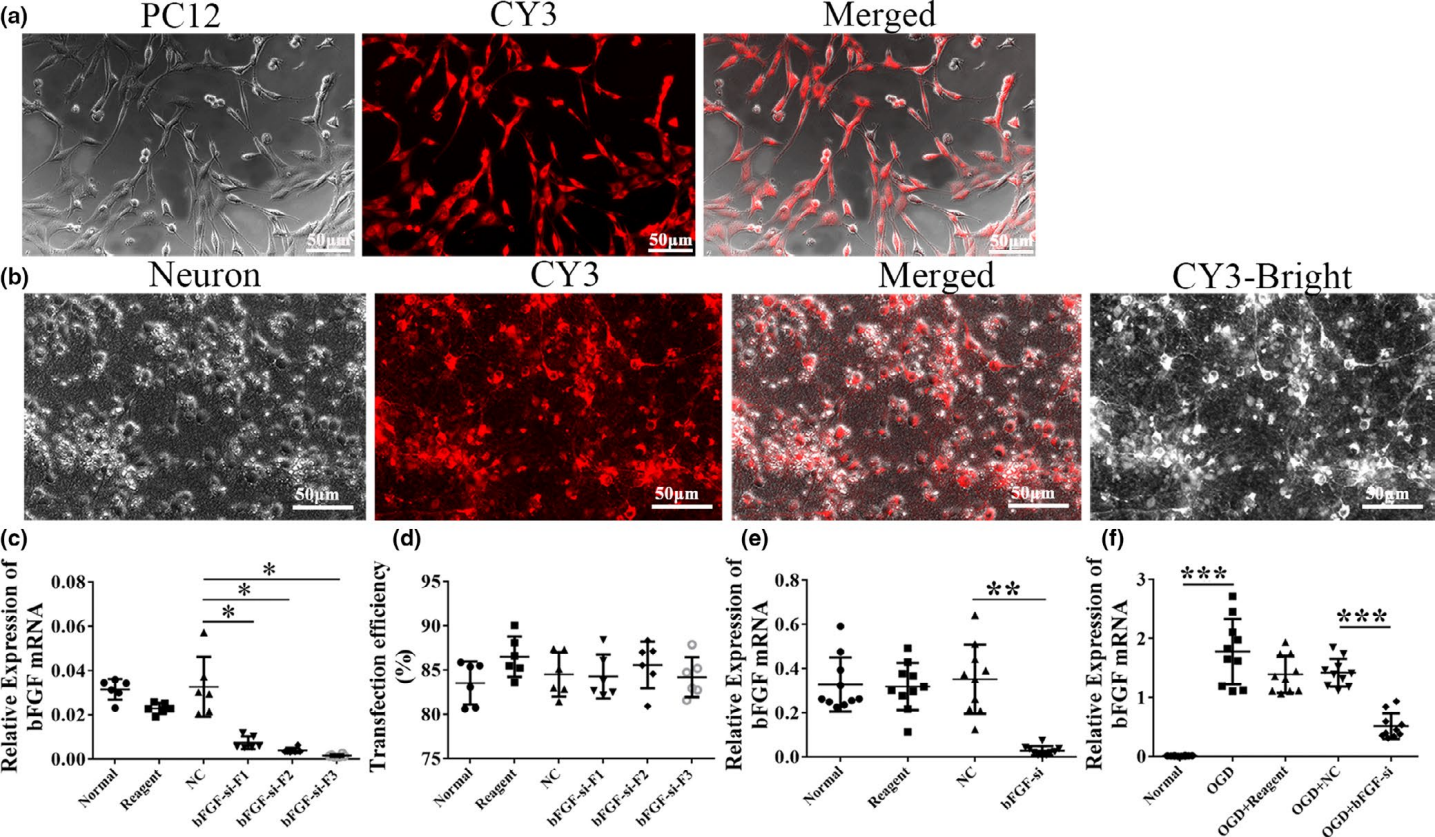
bFGF knockdown was successfully carried out in rat primary cortical neurons. (a) PC12 cells were successfully transfected with bFGF‐F1, bFGF‐F2, and bFGF‐F3 interference fragment by CY3 (red) fluorescent photography. 200×, scale bar = 50 µm. (b) Representative image showing co‐transfection of bFGF‐siRNA/CY3 in rat primary cortical neurons (200×, scale bar = 50 µm). CY3‐Bright: a bright field exposure of CY3 photographs. (c) Quantitative PCR of bFGF was performed among normal, reagent, NC, bFGF‐si‐F1, bFGF‐si‐F2, and bFGF‐si‐F3 groups. The sample size was *n* = 6 for normal, reagent, NC and bFGF‐F1; *n* = 7 for bFGF‐F2 and *n* = 5 for bFGF‐F3 groups. β‐actin served as control for normalization. (d) Quantitative transfection efficiency of bFGF‐siRNA was performed in normal, reagent, NC, bFGF‐si‐F1, bFGF‐si‐F2, and bFGF‐si‐F3 groups. *n* = 6/group. (e) Relative mRNA expression of bFGF among normal, reagent, NC, and bFGF‐si groups. Each group *n* = 10. (f) bFGF mRNA expression was measured among normal, OGD, OGD + reagent, OGD + NC, and OGD + bFGF‐si groups. β‐actin served as control for normalization. *n* = 10/group. Ordinary one‐way ANOVA, with LSD test was performed. *p < .05; **p < .01; ***p < .001. Abbreviation: NC, negative control; bFGF‐si‐F1, bFGF‐siRNA fragment1; bFGF‐si‐F2, bFGF‐siRNA fragment2; bFGF‐si‐F3, bFGF‐siRNA fragment3

Next, we aimed to knock down bFGF in cultured primary cortical neurons to evaluate the role of bFGF in reacting to OGD. We designed three specific siRNA fragments of bFGF, namely F1, F2, and F3, and transfected them into PC12 cells to screen for the most efficient interference fragments (Figure 2a). The results showed that compared with the NC group, the decreased expression levels of bFGF mRNA in the bFGF‐si‐F3 groups were the most significant (*p* < .05, Figure 2c) with a transfection efficiency around 80% in spite of nearly the same transfection efficiency among the three fragments (Figure 2d). Therefore, we selected bFGF‐si‐F3 for the following experiments to transfect primary cortical neurons with a high efficiency suggested by CY3 (Figure 2b). bFGF was successfully knocked down as shown by Q‐PCR (*p* < .01, Figure 2e), whereas reagent group and control siRNA showed no changes of bFGF mRNA compared to normal control. bFGF mRNA levels could be stimulated by OGD condition, which was decreased subject to bFGF‐siRNA knockdown (*p* < .001, Figure 2f). These results suggested that bFGF transcriptional expression could be stimulated by OGD and bFGF‐siRNA could successfully knock down bFGF in primary cortical neuron cultures.

### 3.3 OGD or bFGF knockdown could induce reduction of cell numbers and pathological morphology changes in cultured primary neurons

**Figure 3:**
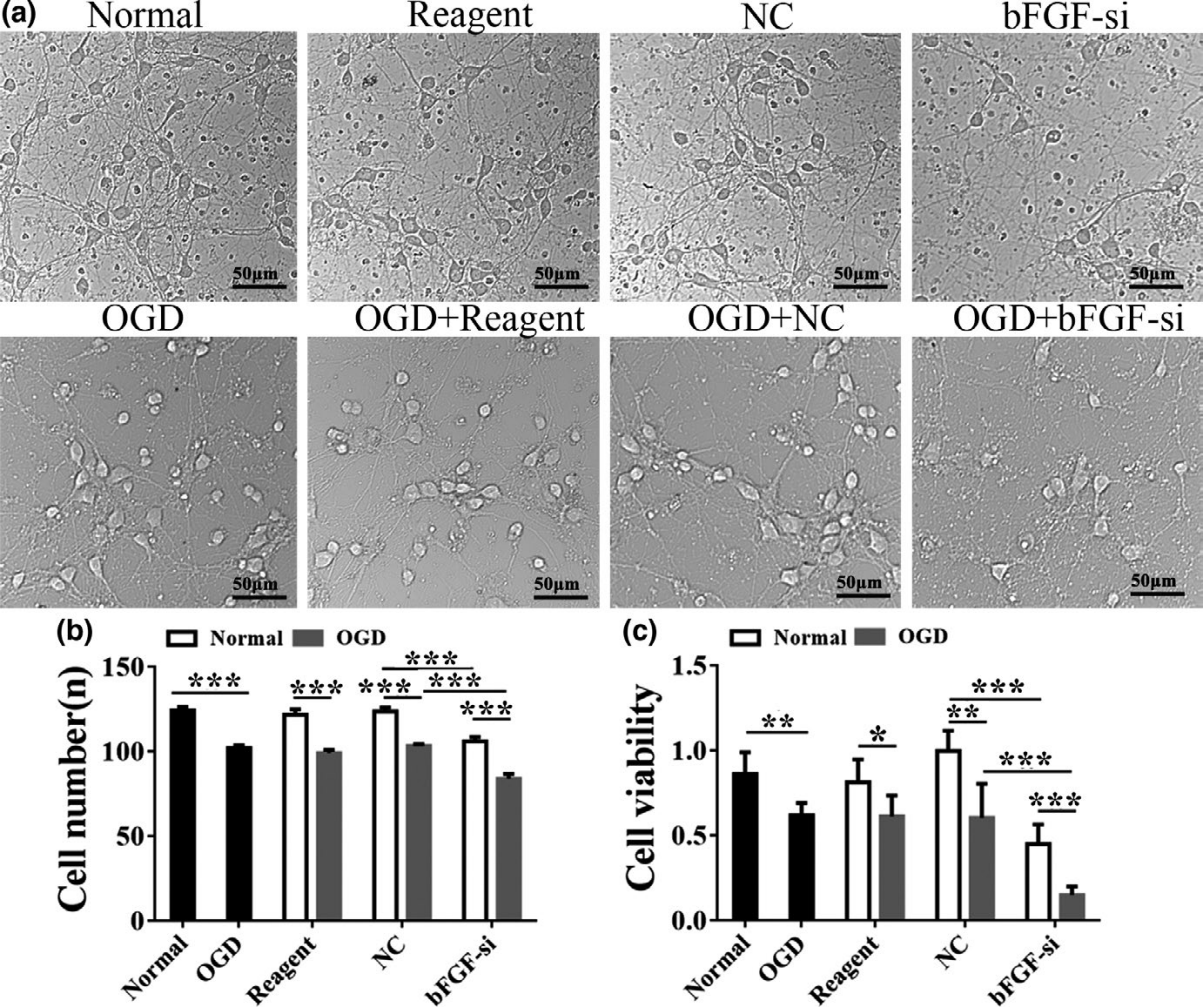
Silencing of bFGF decreased the number of primary cortical neurons after OGD injury. (a) Bright field picture (20 ×) showing morphological changes of neuron among normal group, OGD, reagent group, NC versus bFGF‐si, OGD + reagent and OGD + NC versus OGD + bFGF‐si, respectively. Scale Bar = 50 µm. (b) Quantification of cell numbers among the groups. *n* = 10/group. (c) The viability of neurons was quantified by MTT. *n* = 6/group. Ordinary one‐way ANOVA, with LSD test was performed. ^*^p < .05; **p < .01; ***p < .001

Then, we scrutinized the morphological changes of primary cortical neurons upon bFGF‐siRNA under both normal and OGD settings (Figure 3a). The OGD treatment resulted in more sparse growth of neurons and fewer axons compared to normal control. The reagent and control siRNA had no effects in changing neuronal morphology. Interestingly, application of bFGF‐siRNA under normal culture conditions also gave a similar phenotype of sparse growth, indicating that bFGF might be critical in maintaining normal cell growth of neurons. The combination of OGD + bFGF‐si resulted in more sparse distribution and a more severe morphological phenotype that the cell body of neurons were bulging and broken. To quantify whether the cell numbers were truly reduced in OGD, bFGF‐siRNA, and OGD + bFGF‐siRNA versus controls, we quantified the cell numbers using image processing software. We found that bFGF‐siRNA caused a significant reduction in cell numbers compared to nontargeting siRNA control (NC) (*p* < .001, Figure 3b). Similarly, application of OGD also reduced the cell numbers and OGD + bFGF‐siRNA further reduced the cell numbers of cultured primary cortical neurons (*p* < .001, Figure 3b). Then, the number of cells in the OGD + bFGF‐si group was significantly reduced compared to the bFGF‐si group. After OGD, the number of cells in each group is less than that under normal conditions (*p* < .001, Figure 3b). Cell viability was measured by MTT and found to be the same as the trend of cell number change between these two conditions (Figure 3c). These results suggested that either loss of bFGF or treatment of OGD could cause a decline of cell numbers and cell viability in neurons. Knockdown of bFGF on top of OGD would lead to a worsened phenotype, indicating that bFGF was essential for repairing neuron damage by OGD.

**Figure 4:**
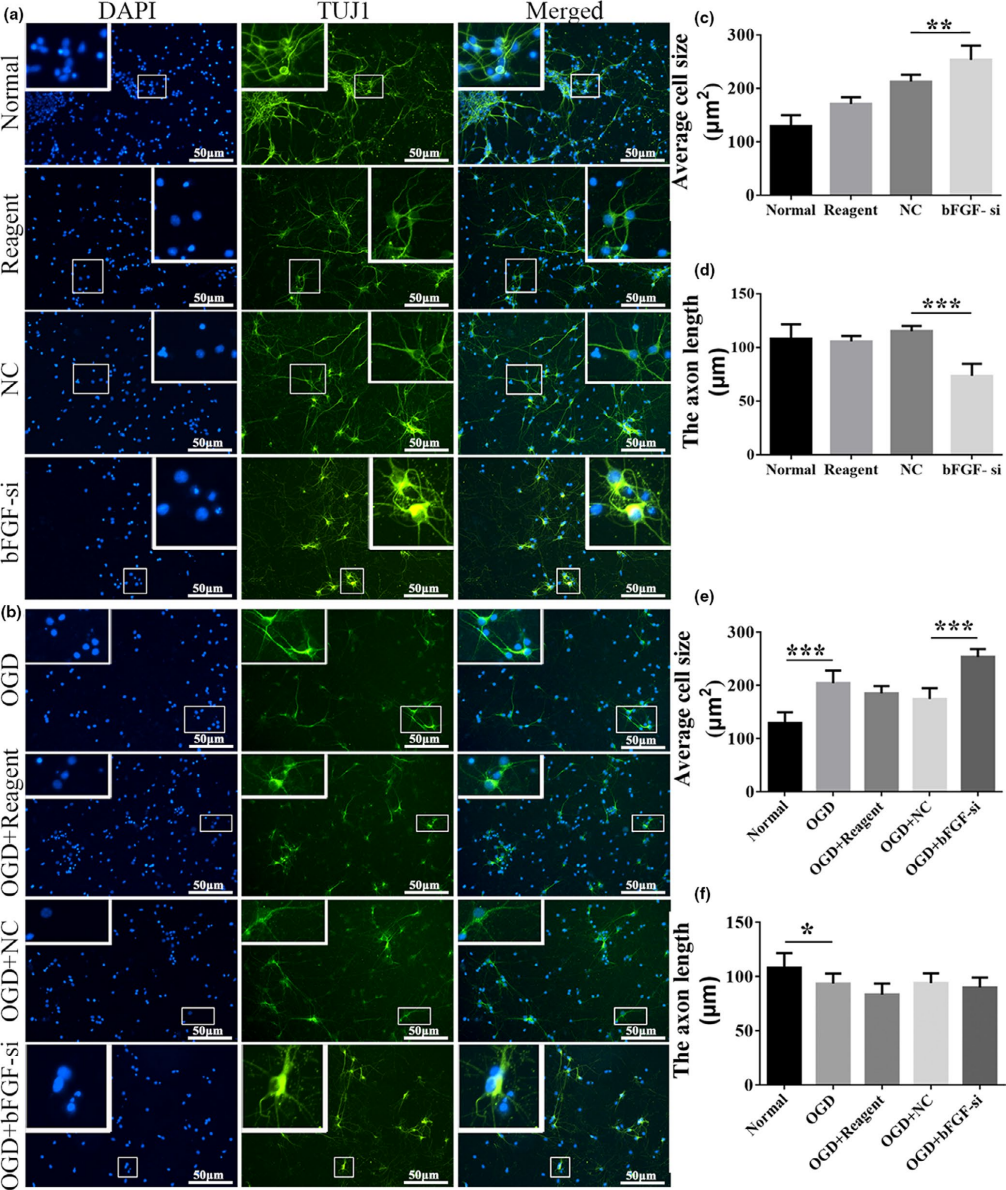
Silencing of bFGF damaged the morphology of cortical neurons and the growth of neuronal axons. (a, b) Immunofluorescence picture (200×, scale bar = 50 µm) showing cell bodies and axons in details. DAPI: nucleus marker (Blue); TUJ1: neuron marker (green). (c, d) The average cell size and the axon length of neurons under normal condition were analyzed by statistics in normal, reagent, NC and bFGF‐si groups. Each group *n* = 6. (e, f) The average cell size and the axon length of neurons under OGD condition were analyzed by statistics in normal, OGD, OGD + reagent, OGD + NC, and OGD + bFGF‐si groups. *n* = 6/group. Ordinary one‐way ANOVA, using LSD test was performed. *p < .05; **p < .01; ***p < .001.

Next, we examined the morphological changes of Tuj1^+^ cells (a classic neuron marker) in details (Figure 4a,b) and quantified the cell body size (Figure 4c,e) and axon length (Figure 4d,f). We noticed that either OGD or bFGF knockdown could generate phenotypes with an enlarged cell body size (Figure 4c,e) and shortened axon length (Figure 4d,f) compared to corresponding controls. Consistent with the cell morphology of bright field image, OGD + bFGF‐siRNA showed exacerbated cell body enlargement, but similar axon length compared to OGD + NC. This could be explained that OGD and bFGF might be involved in synergistic regulatory pathway of axon length. And failure of either was able to shorten the axon length. Taken together, these results showed that either OGD or knockdown of bFGF would cause reduction in cell numbers and pathological morphology alterations, present as enlarged cell body and shortened axon length, in primary cortical neurons. A combination of OGD + bFGF‐siRNA would further reduce the cell numbers and increase the cell body sizes.

### 3.4 IL‐1β expression dynamics in bFGF knockdown, OGD and OGD + bFGF knockdown neurons

**Figure 5:**
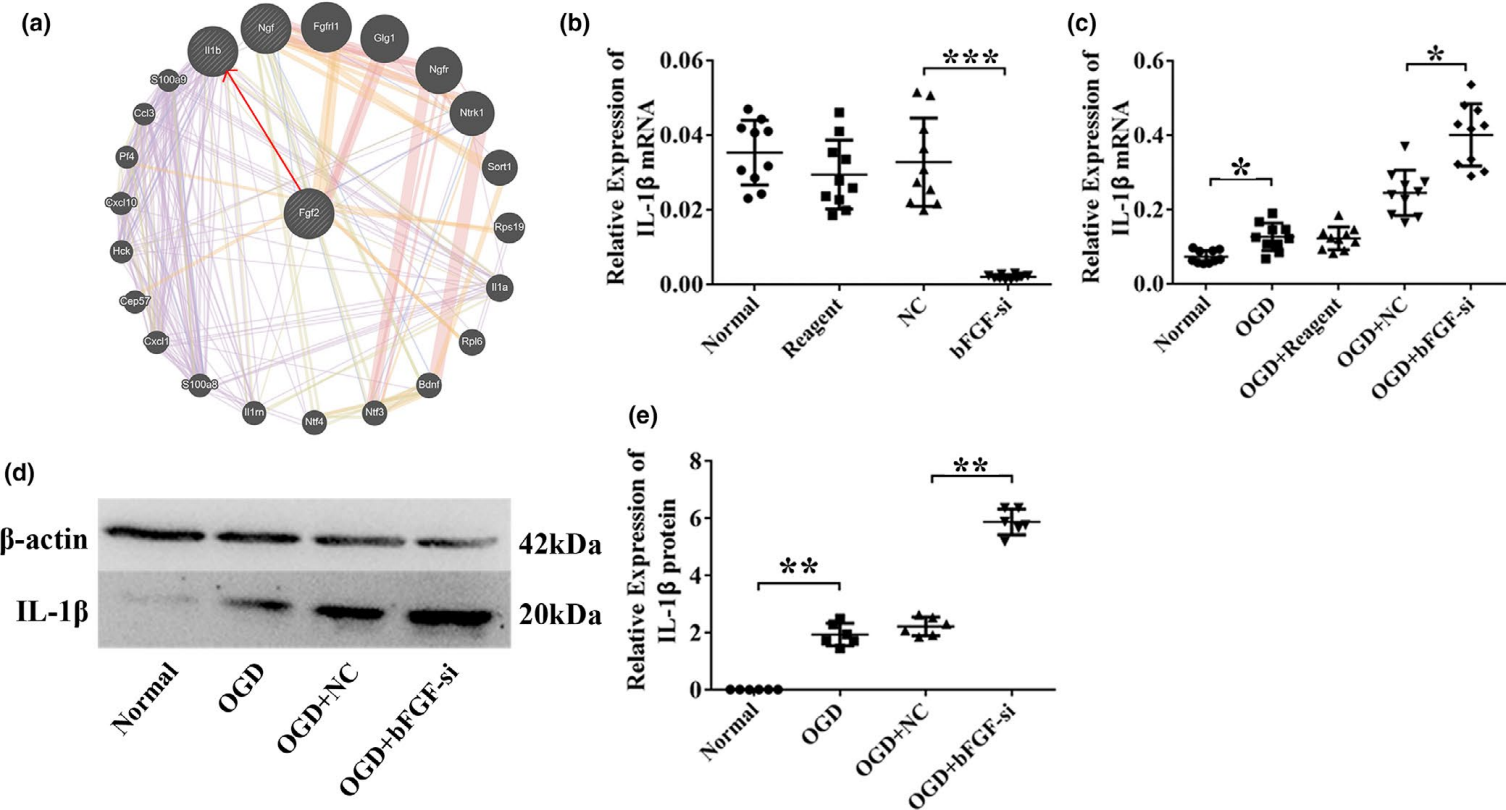
IL‐1β expression dynamics in bFGF knockdown, OGD, and OGD + bFGF knockdown neurons. (a) The bFGF‐related gene network map was predicted by GENEMANIA website. (b–c) IL‐1β mRNA expression was measured in normal, reagent, NC, bFGF‐si, OGD, OGD + reagent, OGD + NC, and OGD + bFGF‐si groups. *n* = 10/group. (d) After OGD induced for 24 hr, representative immunoblots of IL‐1β and β‐actin proteins in the primary cortex neurons of normal, OGD, OGD + NC, and OGD + bFGF‐si groups. (e) Quantitative analyses of IL‐1β protein. Protein loading was calibrated by β‐actin, *n* = 6 for each group. Ordinary one‐way ANOVA, using LSD test was performed. *p < .05; **p < .01; ***p < .001

Because bFGF is a growth factor impacting downstream signaling pathways, we utilized GENEMANIA network station (http://genemania.org/) to identify bFGF targets based on co‐expression patterns (Figure 5a). The top 1 candidate with the most significant co‐expression was IL‐1β. The mRNA expression of IL‐1β was also down‐regulated in bFGF knockdown (*p* < .001, Figure 5b) and up‐regulated in OGD (*p* < .05, Figure 5c). However, when knocking down bFGF under OGD conditions, the expression of IL‐1β was further up‐regulated (*p* < .05). These results were further verified by Western blots (*p* < .01, Figure 5d,e). These results indicated that the regulation of OGD and bFGF on neuron cells was dynamic: IL‐1β expression was closely regulated by bFGF. In order to provide supporting evidence that changes in IL‐1β mRNA levels during bFGF silencing may be involved in the survival of primary cortical neurons under oxygen and glucose deprivation conditions, we investigated the function of IL‐1β by establishing HI models and OGD models.

### 3.5 The changes of neuronal damage and cell morphology in brain tissue after HI by HE and Nissl staining

**Figure 6:**
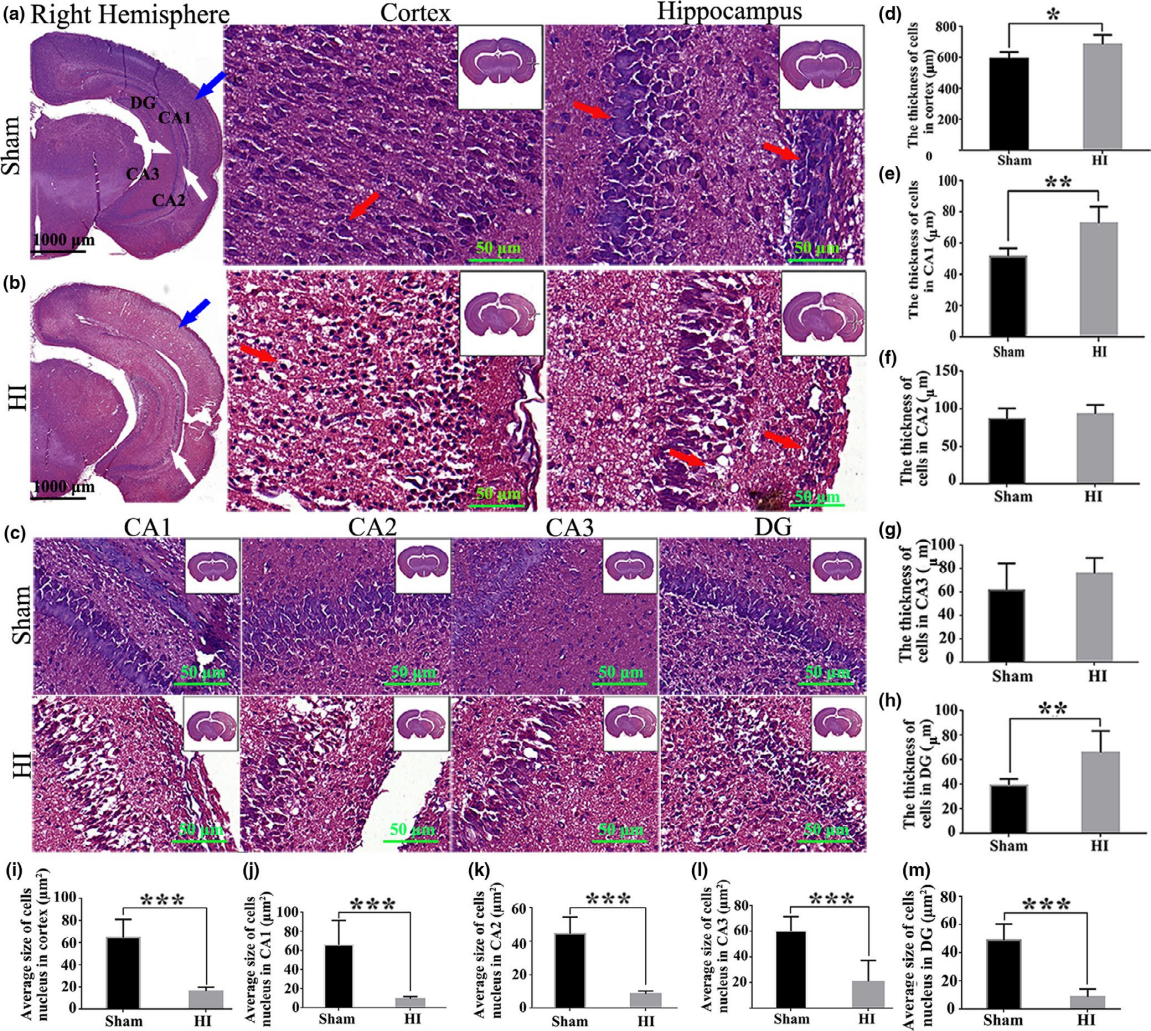
The changes of brain tissue morphology in rats at 24 hours after HI. (a, b) Representative histological section of the cortex and hippocampus was stained by HE staining between Sham and HI groups. From left to right are the sagittal plane (10×, scale bar = 1,000 μm), cortex and hippocampus (200×, scale bar = 50 μm) of the right hemisphere of the brain. The blue arrow represents cortex; the white arrow represent hippocampus; the red arrow represents neurons. (c) Representative histological section of the hippocampal CA1, CA2, CA3,and DG regions in Sham and HI groups was stained by HE staining. Microscope magnification: 200×. Scale bar = 50 μm. (d–h) The thickness of cells in cortical and hippocampal CA1, CA2, CA3, and DG regions was quantitatively analyzed. (i–m) The average size of cell nucleus in cortical and hippocampal CA1, CA2, CA3, and DG regions were quantitatively analyzed. HE, Hematoxylin and eosin; CA, cornu ammonis; DG, dentate gyrus; HI, hypoxia–ischemia. Data are presented as the means ± *SD*. *n* = 8/group. *p < .05; **p < .01; ***p < .001

HE staining was used to observe the morphological variation of neurons from the brain of rats at 24 hr after HI. The results directly showed that there were more cell cavities and the phenomenon of cell nucleus compressed onto one side of neuron cells was emerged in cortex, hippocampal CA1, CA2, CA3, and dentate gyrus (DG) areas after HI as compared with the Sham group (Figure 6a‐c). In addition, quantitative analysis revealed that the thickness of cells in HI group was significantly enhanced in cortex (*p* < .05, Figure 6d), hippocampal CA1 (*p* < .01, Figure 6e), and DG areas (*p* < .01, Figure 6h) compared with the Sham group. Whereas, compared with the HI group, the cell thickness in the hippocampal CA2 and CA3 areas of the Sham group was increased but there was no significant difference (Figure 6f,g). Moreover, the average size of cell nucleus in the cortex and hippocampal CA1, CA2, CA3, and DG areas were markedly smaller in HI group than Sham group (*p* < .001, Figure 6i‐m).

**Figure 7:**
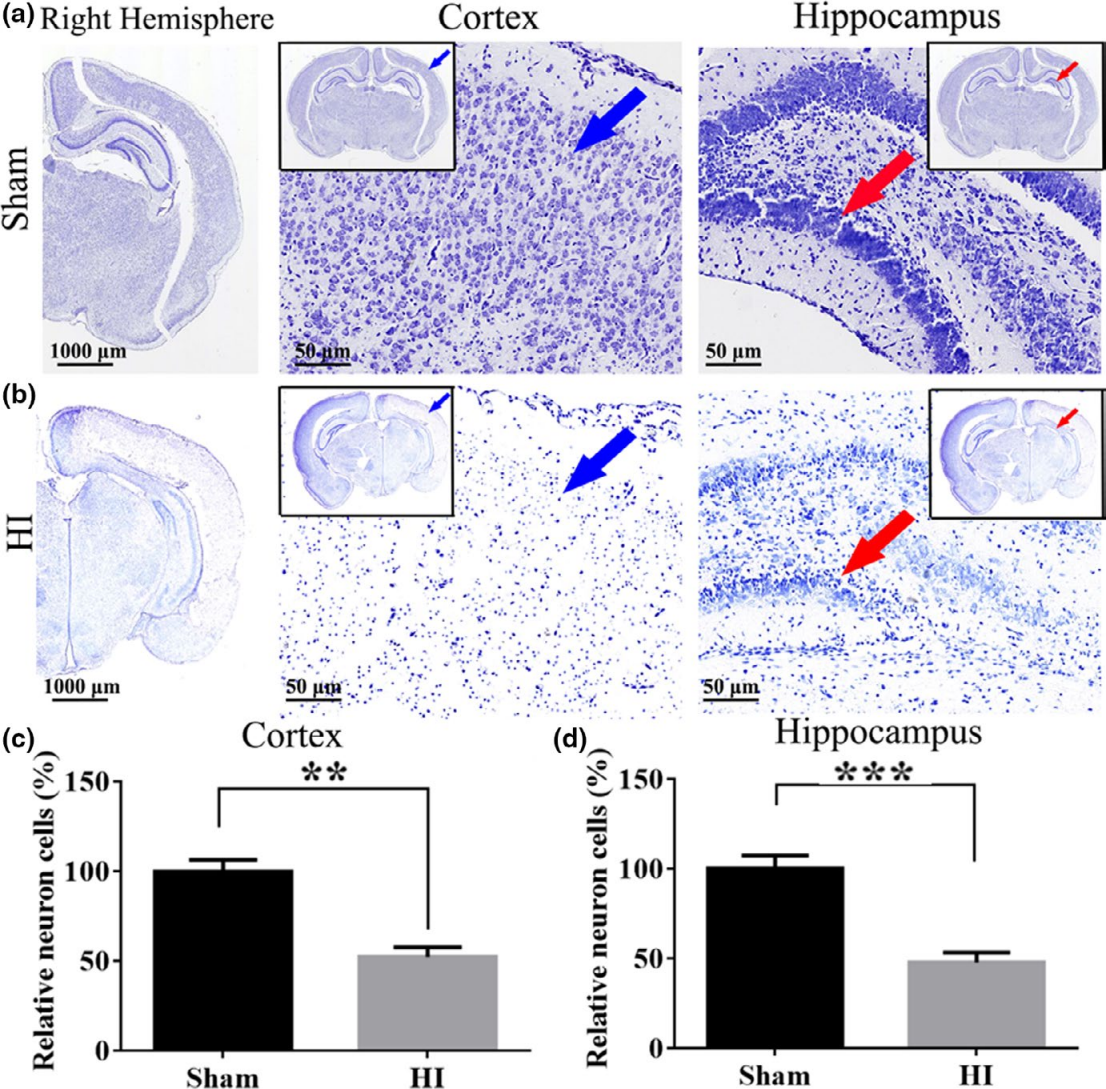
HI induces destruction of cortical and hippocampal neurons. (a) Representative images of Nissl‐stained neurons in the rats cortex and hippocampal DG region of Sham group. From left to right are the sagittal plane (10×, scale bar = 1,000 μm), cortex and hippocampus (200×, scale bar = 50 μm) of the right hemisphere of the brain. (b) Representative images of Nissl‐stained neurons in the rats cortex and hippocampal DG region at 24 hr after HI. The blue arrow represents cortex; the red arrow represents hippocampus. (c–d) The numbers of cortical and hippocampal neurons were quantitatively analyzed. The data are expressed as the mean ± *SD* (*n* = 3/group). **p < .01; ***p < .001. HI, hypoxia/ischemia

To demonstrate the effect of HI on the survival of neurons in the brain, we used Nissl staining to analyze neuronal cell survival in this model. The number of neurons in the cortical (*p* < .01) and hippocampus (*p* < .001) was significantly decreased (Figure 7c,d), the gap was increased, and the volume was decreased in the HI group compared with the Sham group; moreover, the arrangement of neurons was irregular, and nuclear pyknosis was observed in the HI group (Figure 7a,b). The above results prove that the HIE model is successfully established.

### 3.6 Up‐regulation of IL‐1β expression in rat brain tissue after HI

**Figure 8:**
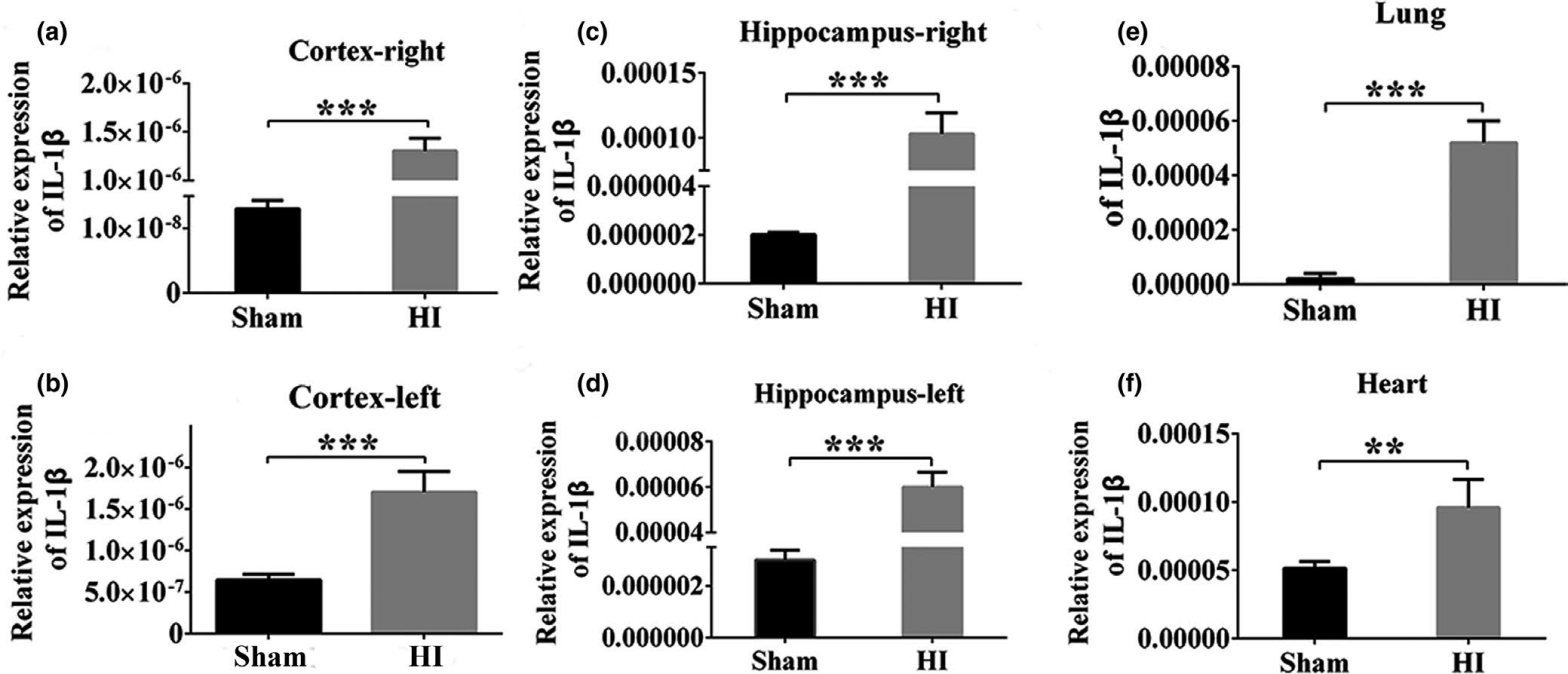
Expression of IL‐1β mRNA in multiple tissues of rats at 24 hr after HI. (a–f) The expression level of IL‐1β mRNA among the right of cortex and hippocampus, the left of cortex and hippocampus, lung and heart tissues were quantitatively analyzed. Data are presented as the means ± *SD* (*n* = 6/group). **p < .01; ***p < .001

The relative expression of IL‐1β mRNA in cortex, hippocampus, lung, and heart tissues was detected by qRT‐PCR. The results showed that IL‐1β mRNA expression in the right of cortex and hippocampus, the left of cortex and hippocampus, lung (*p* < .001, Figure 8a‐e) and heart were increased at 24 hr after HI compared with the Sham group (*p* < .01, Figure 8f). These results are consistent with the changes of IL‐1β expression in primary cortical neurons after OGD, so we constructed IL‐1β‐KO rats to further investigate its function.

### 3.7 Neurological deficits induced by neonatal HI model were improved in IL‐1β KO rats

**Figure 9:**
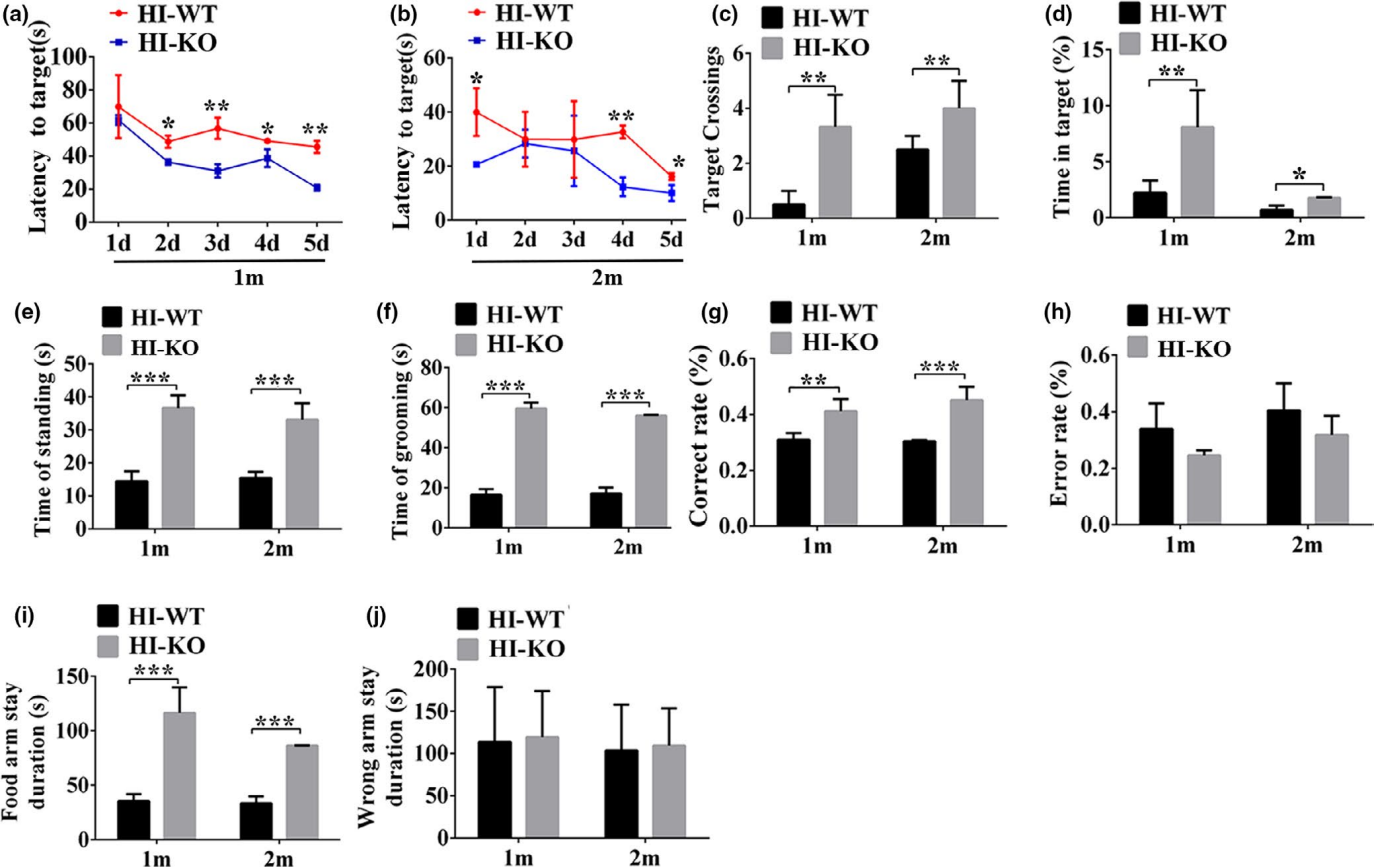
IL‐1β prevents HI‐induced learning and memory impairment. (a, b) Evaluation of escape latency in the MWM test to assess spatial information acquisition. (c, d) Memory capacity is indicated by the number of times the mice crossed the target quadrant and the percentage of time spent in the target quadrant after platform removal on day 6. (e, f) The time spent on standing and grooming in the open field test. (g) The percentage of times that correctly entered the food arm in the Y maze test. (h) The percentage of times that entered the wrong arm in the Y maze test. (i) The time spent on the food arm in Y maze test. (j) The time spent on the wrong arm in Y maze test. Abbreviation: HI‐KO, IL‐1β knockout (KO) rats after HI; HI‐WT, wild‐type rats after HI; HI: hypoxia–ischemia; 1m, one month; 2m, two months. Data are presented as the means ± *SD* (*n* = 8/group). *p < .05; **p < .01; ***p < .001

IL‐1βKO rats were successfully constructed. Animal behavior tests including Morris water maze (MWM), open field test, and Y maze test were performed to further examine the learning, exploratory, and memory functions in IL‐1β KO rats after suffering from HI. The results of a 5‐days directional navigation experiments in MWM showed that the wild‐type rats in HI‐WT groups at one month (1 m) and two months (2 m) after HI took longer to find the hidden platform below the surface of the water than HI‐KO groups (Figure 9a,b). At two months (2 m) after HI, HI‐WT groups still took longer to find the hidden platform below the surface of the water than HI‐KO groups (Figure 9b). After the platform was removed on the 6th day (space exploration experiment), the number of target crossing in the HI‐KO group (*p* < .01, Figure 9c) and the time of stay in the original platform quadrant were significantly increased compared with the HI‐WT group at 1 m and 2 m after HI (*p* < .05, Figure 9d). The results of open field test showed that time of standing and grooming in HI‐KO group were longer than HI‐WT group at 1 m and 2 m after HI (*p* < .001, Figure 9e,f). Furthermore, the results of Y maze test showed that the HI‐WT group had a lower correct rate of finding the food arm than the HI‐KO group at 1 m and 2 m after HI (*p* < .01, Figure 9g). In contrast, the error rate in the HI‐WT group was higher than the HI‐KO group at 1 m and 2 m after HI (*p* > .05, Figure 9h). Moreover, the duration of stay in the food arm (*p* < .001, Figure 9i) and the wrong arm of the HI‐WT group was shorter than that of the HI‐KO group (*p* > .05, Figure 9j). The above results indicate that the learning and memory ability of HI rats was significantly enhanced after knocking out IL‐1β expression.

### 3.8 Knockout of IL‐1β promoted neuronal growth and inhibited neuronal apoptosis

**Figure 10:**
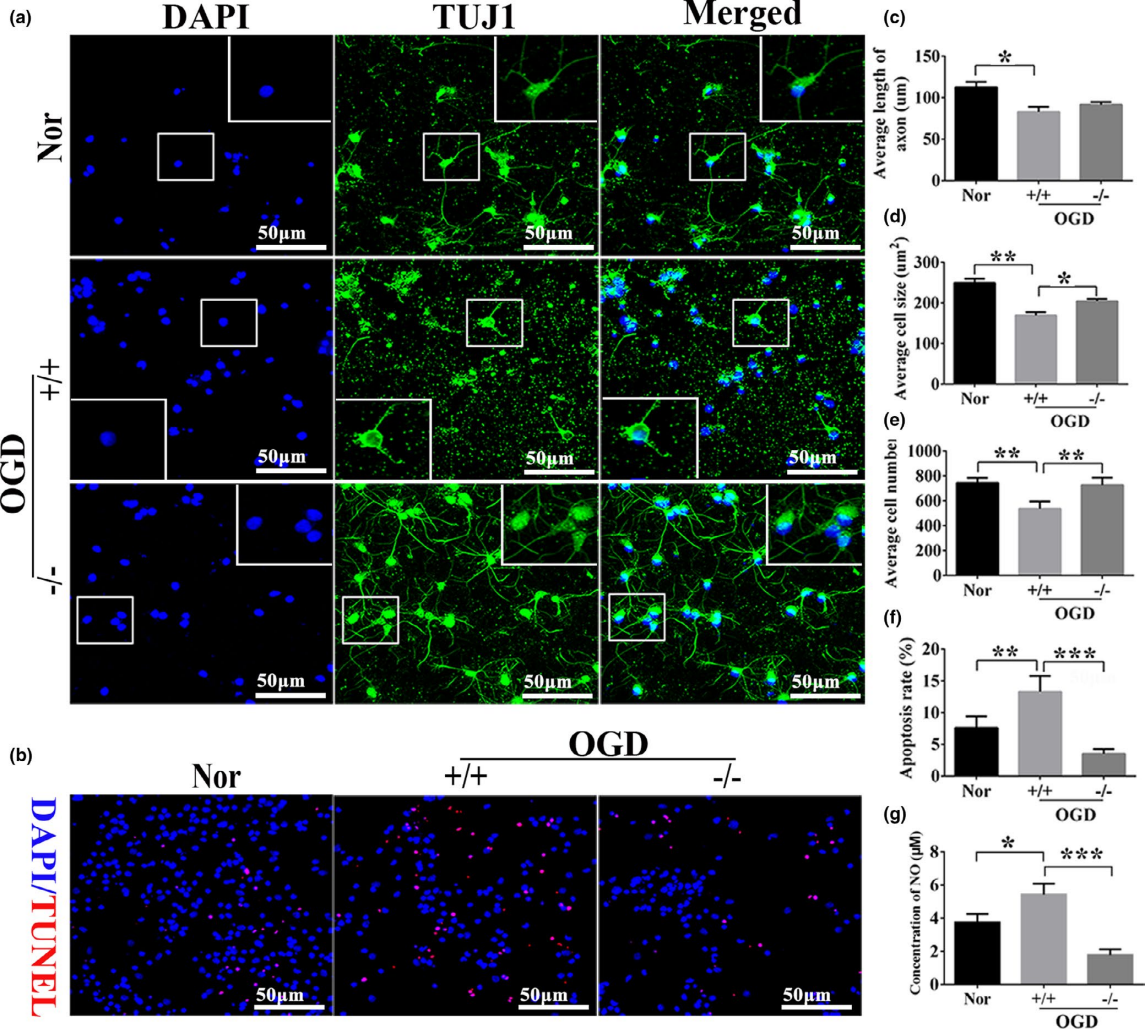
The role of knockout of IL‐1β in the growth of neurons. (a, b) Representative images of immunofluorescent‐stained primary cortical neurons (200×, scale bar = 50 μm). DAPI: nucleus marker (Blue); TUJ1: neuron marker (green); TUNEL: apoptotic cells marker (red). DAPI/TUNEL: nucleus and apoptotic cells merged. (c–f) The bar charts for quantitative analysis of the length of axon, cell body size, the number of cells and cell apoptosis (TUNEL/DAPI (%)) in Nor, +/+ and −/− groups after OGD. (g) The concentration of NO was quantitatively analyzed. Abbreviation: Nor, normal; OGD, oxygen–glucose deprivation; Tuj1, neuronal class III β‐Tubulin; TUNEL, terminal‐deoxynucleoitidyl transferase mediated nick end labeling; DAPI, 4′, 6‐diamidino‐2‐phenylindole; +/+, wild‐type neurons after OGD; −/−, homozygote neurons after OGD. Data are presented as the means ± *SD* (*n* = 8/group). *p < .05; **p < .01; ***p < .001

We carefully observed the morphological changes of Tuj1^+^ cells (classical neuronal markers) (Figure 10a) and quantified the cell body size (Figure 10d), cell numbers (Figure 10e), and axonal length (Figure 10c). Neuronal apoptosis rate and severity of oxidative stress response were quantified by TUNEL (neuronal apoptosis marker) staining and NO (oxidative stress markers) detection (Figure 10b,f,g). Quantitative results showed that the number of cells (*p* < .01, Figure 10e), the length of axons (*p* < .05, Figure 10c), and the cell body area (*p* < .01, Figure 10d) were decreased, and the apoptosis rate (*p* < .01, Figure 10f) and the NO level (*p* < .05, Figure 10c) were increased in the OGD +/+ group compared with the normal group. At the same time, compared with the OGD +/+ group, the cells number (*p* < .01), the axon length (*p* > .05), and the cell body area (*p* < .05) were increased, and the apoptosis rate (*p* < .001) and the NO level (*p* < .001) were decreased in the OGD ‐/‐ group (Figure 10c‐g). The above findings further indicate IL‐1β play a negative role in neuroprotection after OGD.

### 3.9 Up‐regulation of AKT1 expression after IL‐1β knockdown

**Figure 11:**
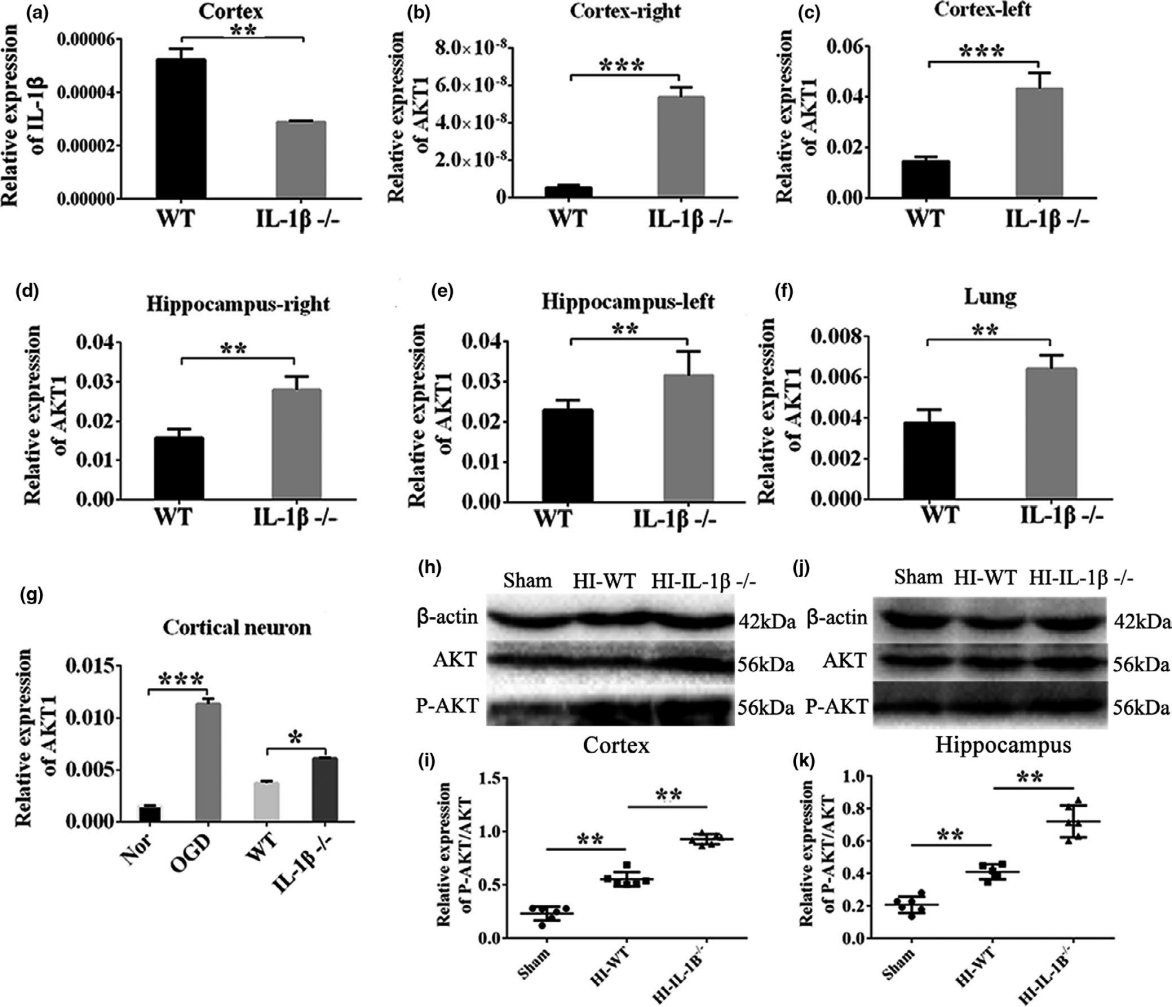
The expression of AKT1 after IL‐1β knockout. (a) The expression level of IL‐1β mRNA in cortex between WT and IL‐1β^−/−^ groups. (b–g) The expression level of AKT1 mRNA among the right of cortex and hippocampus, the left of cortex and hippocampus, lung and primary cortical neurons, was quantitatively analyzed. (h–k) Quantitative analyses of P‐AKT/AKT protein in cortex and hippocampus. Protein loading was calibrated by β‐actin, *n* = 8 per group. Data are presented as the means ± *SD*. *p < .05; **p < .01; ***p < .001. Abbreviation: AKT1, threonine protein kinase; P‐AKT1, phosphorylated AKT; WT, wild‐type rats; IL‐1β^−/−^, IL‐1β knockout (KO) rats; HI‐WT, wild‐type rats after HI; HI‐IL‐1β^−/−^, IL‐1β knockout (KO) rats after HI

To determine whether IL‐1β affects cell growth by modulating AKT1, qRT‐PCR detection was performed in multiple tissues of WT and IL‐1β^−/−^ rats by establishing HI and OGD models. The results showed that the relative expression of IL‐1β mRNA in IL‐1β^−/−^ rat cortex was significantly lower than WT group (*p* < .01, Figure 11a). This result proved that IL‐1β was successfully knocked out. In addition, we found that IL‐1β knockout significantly up‐regulated the expression of AKT1 in rat cortex (right, left) and hippocampus (right, left) as well as lung tissue (*p* < .01, Figure 11b‐f). At the same time, the relative expression of AKT1 mRNA after OGD in primary cortical neurons was much higher than normal group (*p* < .001, Figure 11g). Knockout of IL‐1β significantly increased the relative expression of AKT1 mRNA in cortical neurons compared to the WT group under OGD conditions (*p* < .05, Figure 11g). The above results were further confirmed by Western blots (*p* < .01, Figure 11h‐k). The results suggested that knocking out IL‐1β may improve HI‐induced neurological damage and may be associated with up‐regulation of AKT1.

## 4 DISCUSSION

In this study, we established an in vitro model of hypoxia–ischemic brain damage (HIBD) by treating the isolated rat primary cortical neurons with OGD, mimicking the hypoxia and low glucose supply environment in HI in vivo. We found that either OGD or bFGF knockdown would lead to change of morphology and cell numbers. A combination of both resulted in more reduction in cell numbers and more severe swollen cell bodies of neurons. Also, we identified bFGF relative molecules, IL‐1β that was changed in OGD. To further investigate the function of IL‐1β on neurons, we established IL‐1β KO rats. Our results suggested that bFGF may have a role in promoting neuronal damage and may be associated with down‐regulation of IL‐1β. IL‐1β had a detrimental effect on neurons and may be related to up‐regulation of AKT1.

### 4.1 The implication on the expression of bFGF after OGD

The current study produced a model of OGD in vitro to mimic HIE in vivo (Liu et al., 2018). OGD could up‐regulate bFGF expression, whereas hypoxia could only slightly up‐regulate bFGF. This means that glucose deprivation might play a key role in boosting bFGF in OGD‐treated cells. Studies have found that administration bFGF could protect cells from death (Jungnickel, Klutzny, Guhr, Meyer, & Grothe, 2005; Kusumoto, Dux, & Hossmann, 1997; Lin et al., 2017; Numakawa et al., 2015) and protect the blood–brain barrier (BBB) after intracerebral hemorrhage (ICH) (Huang et al., 2012). Furthermore, the combination of bFGF and other drugs would be a promising therapeutic target for the disease of the nervous system (Lin et al., 2017). In our study, we found the levels of bFGF mRNA in cultured neurons subjected to OGD were increased. We deduced that elevated bFGF might be complimentary response of cells against stress, trying to maintain a normal function of affected neurons.

### 4.2 bFGF plays a neuroprotective role in cultured primary cortex neurons

In the present study, we either knocked down bFGF or administered OGD to induce morphological and cell viability changes of cultured rat neurons. We found OGD could induce swollen cell bodies and shortened axon length, which was consistent with numerous evidence that OGD treatment could deteriorate the growth of neurons (He et al., 2016; Lin et al., 2015; Liu, Yang, Tian, & Ma, 2016; Wang, Liang, et al., 2014; Wu et al., 2018; Zhang et al., 2014) and it could result in neural damage (Kusumoto et al., 1997; Li, Suo, Liu, Li, & Xue, 2017). Further, we confirmed that bFGF‐siRNA had a passive effect on the growth and normal morphology of neurons. Some researchers have proved that bFGF could affect the axonal outgrowth (Bahr, Vanselow, & Thanos, 1989), and up‐regulated bFGF in injury nerve tissue could regenerate axon (Klimaschewski, Nindl, Feurle, Kavakebi, & Kostron, 2004; Lowenstein & Arsenault, 1996). Meanwhile, exogenously administration of bFGF could promote nerve regeneration by strengthening the growth of regenerating axons (Bahr et al., 1989; Lam, Patel, Wang, Chu, & Li, 2010). Barth A et al. have testified that bFGF could attenuate brain injury induced by cerebral ischemia in vitro (Barth, Barth, Morrison, & Newell, 1996). Application of exogenous bFGF in OGD treated neurons should be further performed to evaluate the protective role of bFGF in HI models.

### 4.3 bFGF protected neurons is associated with IL‐1β under OGD condition

In this study, we obtained bFGF‐related molecules in GENEMANIA, which is IL‐1β. Then, we evaluated possible regulatory role of bFGF in fine‐tuning levels of IL‐1β, under OGD or not. IL‐1β was an important mediator of the inflammatory response (Barth et al., 1996). IL‐1β, a member of the interleukin‐1 cytokine family, was activated by macrophages and plays a major role in the development of osteoarthritis, HIE, and liver injury (Aly, Khashaba, El‐Ayouty, El‐Sayed, & Hasanein, 2006; Hong et al., 2013), as well as induces neuronal apoptosis (Fan et al., 2018). It has been reported that oxygen–glucose deprivation and reoxygenation (OGD/R) significantly decreased the cell viability and increased the release of IL‐1β in BV2 microglia cells, and that ginkgolides play a neuroprotective role by reducing IL‐1β expression (Zhou et al., 2016). FTY720 exerts neuroprotective effects in the simulated cerebral ischemia in vitro by reducing the release of IL‐1β (Pang & Hou, 2017). It has also been found that phenoxybenzamine reduced neuronal death in rat hippocampal slice cultures following exposure to OGD by reducing IL‐1β expression (Rau, Kothiwal, Rova, Rhoderick, & Poulsen, 2014). Interestingly, our results showed that silencing of bFGF leads to a decrease of IL‐1β mRNA and to an increase of the mRNA and protein levels in OGD. We hypothesized a two‐way regulation effect of IL‐1β on neuronal survival. There are reports in the literature that IL‐1β promote the survival of neurons and the outgrowth of neurons in culture under normal conditions. IL‐1β may affect as neurotrophic factor in the survival growth and development by indirect mechanism (Kalantari, Ghasemi, Bayani, & Ghaffari, 2016). Based on this result, knockdown of bFGF leads to a decrease of IL‐1β levels under normal conditions, indicating that IL‐1β may have maintenance activity effect on neurons. We could further explore the two‐way regulation effect of IL‐1β on the survival of neurons in the future. Conversely, stimulation of IL‐1β under OGD could be due to inflammatory response of cells which expression was exacerbated when bFGF was absent under OGD, meaning that bFGF might be important in controlling inflammatory reaction in neurons.

### 4.4 Knockdown of IL‐1β improves long‐term cognitive function and promotes neuronal repair in rats after HI

Our data revealed that the neurons were severely damaged in both cortex and hippocampus after HI. Accumulating evidence has indicated that IL‐1β has an adverse effect on neurons after HI injury (Arruza et al., 2016; Kalay et al., 2013; Wang, Zhang, et al., 2014). Moreover, several studies have shown that progesterone and pentoxifylline can induce the IL‐1β expression markedly lower in brain tissue and further reduce brain damage due to hypoxic–ischemic injury (Kalay et al., 2013; Wang, Zhang, et al., 2014). Therefore, in our study, IL‐1β KO rats were constructed by CRISPR/Cas9 technologies were implemented to inhibit the expression of IL‐1β, and it was found that IL‐1β KO rats could notably improve long‐term learning and memory impairment by the test of water maze, open field, and Y maze test at 1 month a 2 month after HI. Meanwhile, neurons growth and decreased apoptosis were more obvious after down‐regulating IL‐1β in OGD model. As we know, the IL‐1β is an inflammatory factor, and the current researches mostly focus on IL‐1β associated with inflammatory reaction and apoptosis. The present study confirmed that novel function of knockout IL‐1β is associated with cognitive function, which significantly affected the long‐term recovery of HIE and presented a better long‐term neurobehavioral outcome after HIE.

### 4.5 Up‐regulation of AKT1 may be one of the mechanisms for improving cognitive function after IL‐1β was knocked out in HIE brain injury

In order to further explore the molecular mechanism of beneficial effects of knockout of IL‐1β after HI, we found that IL‐1β may be closely related to AKT1 through reviewing literature. Several studies have shown that down‐regulation of IL‐1β can exert anti‐apoptotic and anti‐inflammatory effects by activating the AKT pathway (Perez‐Yepez, Ayala‐Sumuano, Lezama, & Meza, 2014; Ren et al., 2017; Su, Peng, Tsai, & Huang, 2013; Tapia‐Abellán, 2014). qRT‐PCR results verified that knocking out IL‐1β could markedly up‐regulate the expression level of AKT1 in multiple area of rats, such as cortex, hippocampus, and lung. It is known that AKT (protein kinase B) is a protein kinase involved in survival signals (Chong, Li, & Maiese, 2005). Evidence in the past few years has shown that AKT pathway was one of the pathways activated by ischemic preconditioning in the adult rodent brain, which may play an important role in ameliorating neuronal survival after ischemia (Yin, Zhang, Miao, & Zhang, 2005). Several studies had indicated that down‐regulation of IL‐1β could activate AKT pathway to produce effect in anti‐apoptosis and anti‐inflammation, and enhancing PI3K/Akt activity was a crucial underlying mechanism (Su et al., 2013). In addition, it was reported that perillaldehyde (PAH) attenuated cerebral ischemia/reperfusion injury in the rat brain cortex, and its neuroprotective effect was related to regulate the inflammatory response through the activation of AKT/c‐JunN‐terminal kinase (JNK) pathway (Xu, Li, Fu, & Ma, 2014). In this study, our data suggested that the down‐regulation of IL‐1β can improve HI‐induced long‐term learning and memory dysfunction. In addition, we further found that the expression of AKT1 was significantly increased after knockdown of IL‐1β under HI condition. Thus, these results revealed that knocking out IL‐1β can improve long‐term cognitive deficits and promote cell growth based on activation of the AKT signaling pathway.

## 5 CONCLUSION

In conclusion, we confirmed that bFGF was essential for maintaining normal cell numbers, viability, and morphology of primary cortical neurons. The induction of bFGF under OGD condition was important for the neurons to handle the stress‐induced damage and may be associated with down‐regulation of IL‐1β. Down‐regulation of IL‐1β could enhance neurons growth and ameliorate the long‐term neurobehavioral recovery, and the underlying mechanism was associated with the up‐regulating AKT1. Therefore, we conclude that IL‐1β‐mediated axonal regeneration is the basis for the mechanism of bFGF in the treatment of HIBD in neonatal rats. The results of this study will provide new insights and molecular basis for clinical therapies for HIBD.

## CONFLICT OF INTEREST

The authors report no conflict of interest.

## AUTHOR CONTRIBUTION

LX, XB, and CB participated in the supervision, guidance of the study, and revision of the paper. ZM was responsible for the design, data presentation, manuscript writing, and revision. FW, JN, and YH cultured primary cortex neurons subjected to OGD, transfected siRNA to neurons, and performed immunofluorescence. ZM and JH performed Q‐PCR and WB experiments. ZN and HW carried out behavioral experiments in IL‐1β KO rats. LX, YS collected data and performed statistics. FW supported this study and revised the paper. All authors have read and approved the final version of the manuscript.

## ETHICS DECLARATIONS

All of the experiments conformed to the Guide for the Care and Use of Laboratory Animals published by the US National Institutes of Health. This study was conducted in accordance with the principles of the Basel Declaration and recommendations of the Ethical Committee of Kunming Medical University (reference number: kmmu 2018024).

## Supporting information

Figure S1

## Data Availability

The data supporting the results of this study are publicly available.
