# Corrigendum to bFGF promotes neurological recovery from neonatal hypoxic–ischemic encephalopathy by IL‐1β signaling pathway‐mediated axon regeneration

**DOI:** 10.1002/brb3.2548

**Published:** 2022-04-18

1


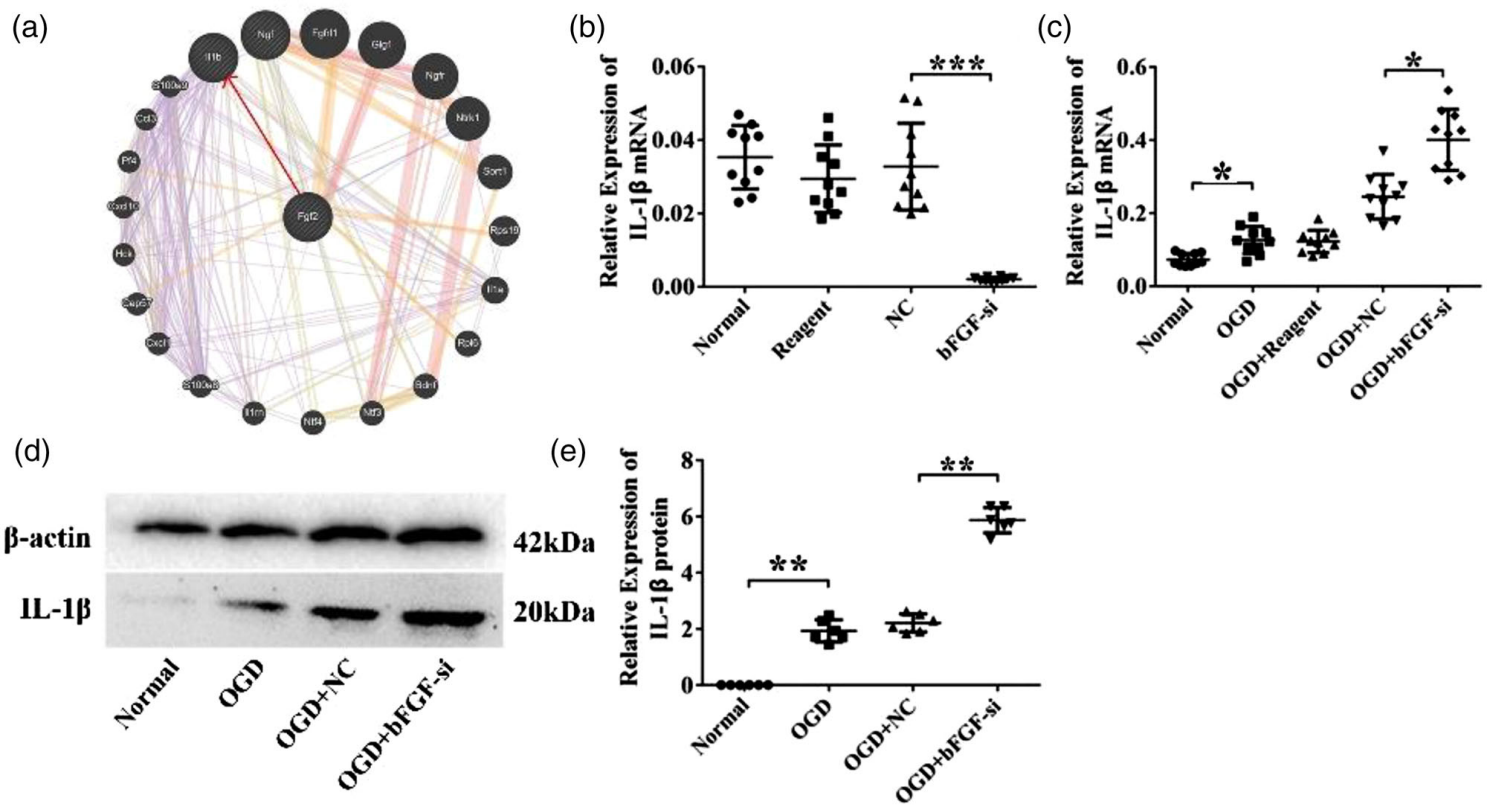


Ma Z, Wang F, Xue L‐L, et al. bFGF promotes neurological recovery from neonatal hypoxic–ischemic encephalopathy by IL‐1β signaling pathway‐mediated axon regeneration. *Brain Behav*. 2020;10:e01696. https://doi.org/10.1002/brb3.1696

The authors regret some clerical errors made in the Materials and Methods section regarding secondary antibodies. In Section 2.22, the following error was published as:

After incubation with appropriate secondary antibodies (HRP, Goat Anti‐Mouse IgG, cat# A21020, 1:5,000; HRP, Goat Anti‐Mouse IgG, cat# A21010, 1:5,000) for 1 hr at room temperature, protein bands were visualized and analyzed using a chemiluminescent imaging system (Bio‐Rad).

The text was incorrect and should have read:

After incubation with appropriate secondary antibodies (HRP, Goat Anti‐Mouse IgG, cat# A21010, 1:5,000; HRP, Goat Anti‐Rabbit IgG, cat# A21020, 1:5,000) for 1 hr at room temperature, protein bands were visualized and analyzed using a chemiluminescent imaging system (Bio‐Rad).

In Figure 5D, blurry bands of β‐actin have been substituted with more clear ones. The corrected figure appears here:

