# Supplementary figures and images for: bFGF promotes neurological recovery from neonatal hypoxic–ischemic encephalopathy by IL‐1β signaling pathway‐mediated axon regeneration
Source: Brain Behav. 2020 Jun 11;10(8):e01696. doi: 10.1002/brb3.1696 (PMC7428497; doi:10.1002/brb3.1696)

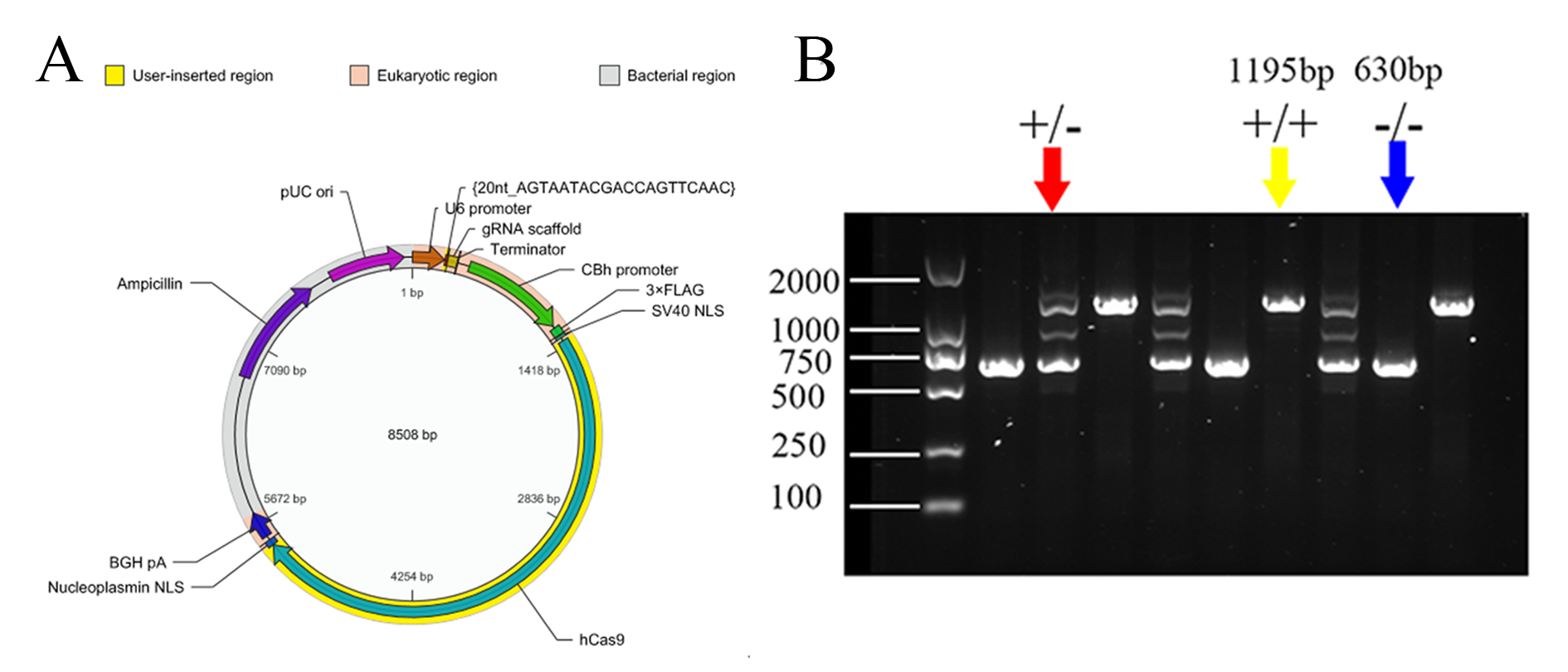

Supplement: Supplementary file 1 — Figure S1 [file BRB3-10-e01696-s001.tif]
